# Multiresidue Methods Analysis to Detect Contamination of Selected Metals in Honey and Pesticides in Honey and Pollen

**DOI:** 10.3390/foods13244099

**Published:** 2024-12-18

**Authors:** Mattia Casula, Francesco Corrias, Alessandro Atzei, Massimo Milia, Nicola Arru, Alberto Satta, Ignazio Floris, Michelina Pusceddu, Alberto Angioni

**Affiliations:** 1 Food Toxicology Unit, Department of Life and Environmental Science, University Campus of Monserrato, University of Cagliari, SS 554, 09042 Cagliari, Italy; mattiacasula92@gmail.com (M.C.); francesco.corrias@unica.it (F.C.); max_milia@hotmail.it (M.M.); nicola.arru.logica@gmail.com (N.A.); 2 Department of Agricultural Sciences, University of Sassari, Viale Italia 39A, 07100 Sassari, Italy; albsatta@uniss.it (A.S.); ifloris@uniss.it (I.F.); mpusceddu@uniss.it (M.P.)

**Keywords:** industrial processing, honeybee, LC-MS/MS, pesticide residues, heavy metal and metalloid, ICP-OES

## Abstract

Honey, a natural food with a rich history, is produced by honeybees and other species of bees from nectar, other plant fluids, and honeydew of sap-sucking insects. During foraging, these bees may be exposed to plant protection products (PPPs), metals, and metalloids, potentially leading to residues in honey and hive products that could have a negative impact on human safety. Recognizing the lack of an appropriate methodology for pesticide contamination of honey and other hive products, this research aims to support the need for studies on residues in pollen and bee products for human consumption to establish safe maximum residue levels (MRLs) for consumers. A UHPLC-MS/MS residues method and a modified QuEChERS extraction were applied to simultaneously determine 237 pesticide residues in honey and pollen. The study in North Sardinia analyzed honey and pollen samples from six areas for pesticide residues and verified 27 heavy metals and metalloid residues using Inductively Coupled Plasma Optical Emission Spectrometry (ICP-OES). The data obtained showed residues at levels close to the LOQ of the method, cycloate in a few samples of pollen, and dichlorvos, zoxamide, cycloate, and chlorantraniliprole in honey samples. All samples showed the absence of heavy metal contamination. Overall, no risk to human health was identified. The results of this study confirm that honey and pollen may be a good bioindicator of environmental contamination of a wide area surrounding honeybee hives.

## 1. Introduction

Honey and pollen, with their rich history dating back to ancient times, have always held a special place in human culture. They were considered by the Greeks to be the food of kings and were used by Sumerians and Egyptians for medicine and religious purposes [1,2]. The *Codex Alimentarius* defines honey as a natural substance produced by honeybees (*Apis mellifera*) from the nectar of plants, secretions of living parts of plants, or excretions of sup-sucking insects. Honeybees collect and transform the nectar by combining it with specific substances and then storing it in honeycombs to mature [3,4]. Bee honey, a unique and complex viscous solution, is rich in various compounds, such as sugars, water, minerals, proteins, amino acids, and low levels of enzymes, vitamins, and phenolic compounds [4,5]. Its composition, a result of the intricate interplay of floral sources, its geographical origin, seasonal and environmental factors, and processing methods, gives it a range of unique properties [6]. Bee honey and pollen have been used in many medical applications for their antioxidant, anti-inflammatory, antibacterial, and anticarcinogenic properties, making them a fascinating subject of study [7]. Processing the nectar of flowers into honey is a complex work that requires collaboration in the bee colony. A honeybee colony comprises one queen bee, i.e., a unique fertile female, a few thousand drone bees, i.e., fertile males, and thousands of worker bees, i.e., sterile females [8]. The worker bees perform specialized roles as foragers and house bees in honey production. Drone bees are bred during the breeding season, which starts with the construction of comb cells [8]. Honey production begins when foragers fly out from the hive to collect nectar from flowers. Nectar is the main raw material used in honey production. Not all flowers produce nectar, and not all are accessible to honeybees. Moreover, the amount and concentration of the nectar change from plant to plant and over time [8]. Using their proboscis, foragers collect the nectar from flowers and store it in a particular organ called the honey sac: an enlargement of the esophagus separated by a valve from the rest of the digestive tract. Bees can visit from 50 to 100 flowers before returning to the hive, and during the return journey, they begin to reduce the water content of the nectar solution through evaporation [9]. Honey production continues in the hive when multiple worker bees share nectar among nestmates (trophallaxis) and evaporate the nectar to reduce water content further. Worker bees add microbes like lactic acid bacteria derived from the gut to digest complex polysaccharides during this process. They also add enzymes like invertase (D-fructo-furanosi-d-fructohydrolase) that hydrolyze sucrose in fructose and glucose. In addition to nectar, honeybees gather pollen from flowers, which fulfills the colony’s need for protein, minerals, and fats [10]. Unlike nectar foraging, which is primarily influenced by availability, pollen foraging behavior is extremely sensitive to the colony’s condition. Specifically, eggs and young and old larvae stimulate pollen foraging [11,12]. Inside the hive, pollen is fermented into bee bread, which contains essential amino acids required for honeybee development and can be stored in the nest for a prolonged period [13,14,15,16,17]. During the collection of nectar and pollen, honeybees may be exposed to plant protection products (PPPs), metals, and metalloids. Honey and hive products can potentially contain residues that could be harmful to human health [18,19], and honeybees can be used as bioindicators of contaminants in the environment [20,21,22,23]. In addition to the effects on human health, pesticides can negatively affect the honeybee, causing deformity in the ovary size of the queen, lower sperm quantity, and sperm viability in the spermathecae (thiamethoxam and clothianidin) [24]. Furthermore, several classes of pesticides negatively impact bee growth and development and reduce their foraging activity and pollination services, affecting their olfactory senses, memory, navigation to the nest, flight ability, and dance communication [25]. The OECD has a guide for quantitatively assessing the adverse effects of plant protection products on the honeybee brood under conditions close to the real world [26]. Regulation CE 37/2010 sets the maximum residual levels (MRLs) for amitraz at 0.2 mg kg^−1^ and coumaphos at 0.1 mg kg^−1^ [27]. However, the study of pesticide contamination of honey and other hive products lacks a proper methodology. Therefore, pesticide MRLs for honey have been historically set at a default level of 0.05 mg kg^−1^. To fill this gap, the SANTE technical guide stipulates the circumstances under which MRLs in honey should be considered established for a given compound [28]. EFSA PRIMo (the Pesticide Residue Intake Model) states that even the default value of 0.05 mg kg^−1^ should be checked in terms of acute risk of dietary exposure. This work is intended to provide complementary and new knowledge to the already existing knowledge on honey and honey products as indicators of environmental contamination. Moreover, it aims to increase the knowledge of the content of pesticide residues in honey and pollen obtained by honeybees. A UHPLC-MS/MS residues method with modified QuEChERS extraction was validated to determine 237 pesticide residues in honey and pollen. Honey and pollen samples collected from six areas of North Sardinia were analyzed for pesticide residues. Moreover, 27 heavy metals and metalloid residues were verified by Inductively Coupled Plasma Optical Emission Spectrometry (ICP-OES). Cossu et al. (2003) evaluated the possible contamination of fenpropathrin, cyfluthrin, deltamethrin, and permethrin residues in 74 honey samples produced in Sardinia during 2000–2001 [29]. Elements such as Fe, Mn, Cu, Zn, and Ni are essential for the physiology of the human body, but elevated levels of these elements can be dangerous to human health. High nickel, cadmium, and lead have carcinogenic and cytotoxic properties [30]. Massidda et al. (2007) and Satta et al. (2012) studied the contamination of As, Cd, Cu, Mn, Ni, Pb, V, and Zn, and Cd, Cr, and Pb from different sites in different areas of the southwest of Sardinia by ICP mass and electrothermal atomic absorption spectrometry, respectively [31,32]. No other studies dealing with the use of honey and other beehive products as an indicator of the environmental contamination in Sardinia are present in the literature.

## 2. Materials and Methods

### 2.1. Chemicals and Reagents

The acetonitrile (ACN) and methanol (MeOH) used were LC/MS grade solvents (Sigma Aldrich, Milan, Italy). The formic acid used was reagent grade (>95%, Honeywell, Sigma Aldrich, Milan, Italy), ammonium formate solution 5 M (0.315 g mL^−1^) (G1946-85021, Agilent Technologies, Milan, Italy). QuEChERS reagents Part No: 5982-6650 (4 g MgSO_4_, 4.1 g NaCl, 1 g trisodium citrate dihydrate, 0.5 g disodium hydrogen citrate sesquihydrate; En Method 15662), and Part No: 5982-5056 (150 mg PSA, 900 mg MgSO_4_; EN Method, fruit and vegetable) were purchased from Agilent Technologies (Milan, Italy). MilliQ water with a conductivity of less than 18.2 MΩ was obtained from an integrated Millipore purification system (MilliQ integral, Merck, Milan, Italy). Certified analytical standards (≥98.0% purity) of 237 pesticides at 100 mg L^−1^ in ACN (LC/MS Pesticide Comprehensive Test Mix Kit–Part number: 5190-0551) were purchased from Agilent Technologies (Table S1). The intermediate solution of the pesticide mix was prepared at 1 mg L^−1^ in ACN + 0.1% formic acid. The five-point matrix-matched calibration curves were prepared daily by serial dilution of the intermediate solution in blank honey and pollen extract (10 mL). HNO_3_ (67–69%), 30% H_2_O_2_ solution and standards of Ag, Al, AS, B, Ba, Be, Ca, Cd, Co, Cr, Cu, Fe, Hg, Li, Mg, Mn, Mo, Ni, Pb, Sb, Se, Sn, Sr, Te, Ti, V, and Zn were of ICP grade (Carlo Erba Reagents, Milan, Italy). The HCl (34–37%) used was of super-pure quality (Romil Spa, Cambridge, UK).

### 2.2. Samples Collection and Preparation

Samples were collected from six sites: four in the North-West (NW) Sardinia: Codrongianos (ϕ 40°41′14.50″; λ 8°40′00.20″; MSL 275 m), Florinas (ϕ 40°30′20.06″; λ 8°30′11.03″; MSL 297 m), Ittiri (ϕ 40°32′34.55″; λ 8°34′59.19″; MSL 409 m), and Villanova Monteleone (ϕ 40°39′28.60″; λ 8°37′32.65″; MSL 444 m); and two in North-East (NE) Sardinia in La Maddalena Archipelago, or more precisely, La Maddalena (ϕ 41°13′23.6″; λ 9°23′46.8″; MSL 150 m), and Caprera (ϕ 41°12′59.4″; λ 9°28′15.6″; MSL 212 m). All sites were characterized by a similar complex plant ecosystem composed of annual and perennial plant species. Among the 29 species identified, Fabaceae (27), Asteraceae (15), and Caryophyllaceae (5) were the most abundant. In these environmental conditions, honeybee activity is typically concentrated within 1–2 km from the apiary [33]; therefore, to ensure site independence, the areas were selected between a 5 (Codrongianos–Villanova) and 25 km distance (Codrongianos–Florinas), and the sites of La Maddalena and Caprera ensured complete independence of the apiaries. At each sampling site, four honeybee colonies were placed in a small apiary on private land. The collection of honey and pollen samples started after a 5-month acclimatization period of the hives at the selected sites. Unripe honey samples were collected by recovering them with a 50 mL syringe from 100 unsealed cells from three nest combs [34]. For pollen sampling, each honeybee colony was equipped with a pollen trap placed at the hive’s entrance and activated 48 h before the sampling date to ensure sufficient and more representative pollen collection [35]. Honey and pollen samples were collected once a month from each honeybee colony in the following period: from June 2020 to November 2020 and from March 2021 to September 2021 in the sites of La Maddalena and Caprera; from February 2021 to October 2021 in the sites of Codrongianos, Florinas, Ittiri, and Villanova. During the collection period, each hive was monitored every 10 days approximately to check for the presence of the queen, brood, and food stores. If the check on the honeybee colony revealed that the queen was missing, and swarming was in progress or had just occurred, samples were not collected.

#### 2.2.1. Sample Preparation for Pesticide UHPLC-MS/MS Analysis

Samples were prepared for LC-MS/MS analysis according to Anastasiades and Lehotay (2003) [36]. One gram of honey or pollen was weighed in a 15 mL test tube with 1 mL of water and warmed at 30 °C in a bain-marie for 1 min. Subsequently, 5 mL ACN was added and agitated in the vortex for 1 min (Reax Top, Heidolph, Germany). After that, 0.65 g of QuEChERS salts (Part No: 5982-6650) was added, and the test tube was agitated for 2 min in the vortex and 15 min in a rotatory shaker. The sample was centrifuged for 5 min at 3154× *g* and 10 °C (Centrifuge 5810 R, Eppendorf AG 22331 Hamburg). Then, the supernatant was recovered and transferred to a 15 mL test tube containing 1 g of the second QuEChERS salts (Part No: 5982-5056, Agilent, Milan, Italy). The tube was agitated in a vortex for 2 min and then in a rotatory shaker for 15 min; the solution was centrifuged for 5 min at 3154× *g* at 10 °C. The organic solution was filtered at 0.45 µm (PTFE, Thermo Scientific, Milan, Italy) and transferred to a 1.8 mL vial for LC-MS/MS analysis.

#### 2.2.2. Sample Preparation for ICP-OES Analysis

Approximately 0.5 g of honey was weighed and digested with 1 mL of HNO_3_/HCl solution 1:3 (*v*/*v*) and 1 mL of 30% H_2_O_2_ in closed polytetrafluoroethylene (PTFE) tubes using a CEM MARS 6 microwave digestion system (CEM SRL, Modena, Italy). A three-stage protocol was used as follows: heating time: 2 min; pressure: 100 PSI; and power: 300 W. After digestion, the solution was left to cool at room temperature, placed in a 10 mL flask, diluted with H_2_O at 1% HNO_3_, and filtered through a 0.45 μm nitrocellulose membrane filter. Control solvent samples were prepared during heavy metal and metalloid analysis to avoid false positives and contamination. 

### 2.3. UHPLC-MS/MS Analysis

A UHPLC Agilent 1290 Infinity II LC (Agilent Technologies, Milan, Italy) coupled with an Agilent 6470 Triple Quad LC-MS/MS mass detector (Agilent Technologies, Milan, Italy) with a MassHunter ChemStation (Agilent Technologies, Milan, Italy) was used. The column was a ZORBAX Eclipse Plus C18 (2.1 × 150 mm, 1.8 μm). A binary gradient of H_2_O 5 mM in ammonium formate + 0.1% formic acid (A) and methanol 5 mM in ammonium formate + 0.1% formic acid (B) was set as follows: t = 0 A 95%; t = 1 min A 95%; t = 3.00 min A 55%; t = 16 min A 5%; t = 22.50 min A 5%; t = 22.60 min A 95%; with a post-run of 6 min (95% A). The total duration of the chromatographic run was 28.60 min, and the flow was 0.3 mL min^−1^, with 1 μL of the sample volume injected in positive mode. Mass detector gas and sheath gas were set at 120 °C and 350 °C, respectively, with a gas flow of 10 L min^−1^, a sheath-gas flow of 12 L min^−1^, nebulizer 30 psi, positive capillary of 4000 V, and dynamic MRM (Table S1). Calibration curves were calculated with five points starting from the LOQ value and were considered acceptable when r^2^ ≥ 0.995 (Table S2).

### 2.4. ICP-OES Analysis

Agilent 5100 Inductively Coupled Plasma Optical Emission Spectrometry (ICP-OES, Agilent Technologies, Santa Clara, CA, USA) was used to determine trace metals in the honey samples. The working conditions were 1200 W for the radio frequency (RF), a 0.7 L min^−1^ nebulizer flow, a 1.00 L min^−1^ auxiliary flow, a 12.0 L min^−1^ plasma flow, a 12 rpm pump speed, axial plasma viewing, and a read time of 5 s. Detection was performed at the specific emission wavelengths (Table S3). As, Hg, Sb, and Se sample preparation was carried out using an Agilent VGA-77 (Agilent, Milan, Italy) according to Agilent application notes [37,38]. Calibration curves were calculated with five points ranging from 0.005 mg kg^−1^ to 1.00 mg kg^−1^ and were considered acceptable when r^2^ ≥ 0.995. 

### 2.5. Method Validation

The analytical methods were validated according to the SANTE Guidelines (SANTE 2021) by evaluating linearity, the limit of quantitation (LOQ), accuracy (expressed as recovery percentage), precision (% relative standard deviation), ruggedness, and selectivity [39]. Blank control samples were collected from appropriately selected organic hives. The analytical LOQ was set at the minimum concentration of each analyte in the matrix samples, providing suitable recovery and precision data (Supplementary Table S1). Six blank control honey and pollen samples were spiked with the mixed multiresidue standard at 5 × LOQ and analyzed in one day for repeatability (RSDr, intraday n = 12). In comparison, reproducibility (RSDwR) was calculated by analyzing two samples at 5 × LOQ for each matrix for six days (n = 12). Each sample belonged to an independent experiment. Control blank samples for recovery assays were fortified at LOQ and 10 × LOQ with the mixed multiresidue pesticide standard and left standing for 30 min. Three replicate samples of each concentration were analyzed for each matrix (n = 12). Recovery results were analyzed using matrix control standard calibration curves. The instrumental sequence was conducted according to SANTE indications. The matrix effect was evaluated by comparing the analytical responses of the active ingredients in ACN + 0.1% formic acid with those prepared with blank control matrix extracts. Linearity was assessed by analyzing five standard calibration curves performed in triplicate. Calibration curves were prepared in ACN + 0.1% formic acid and blank control matrix extracts (honey and pollen). They were admitted as acceptable when the coefficient of determination was above 0.995. Selectivity was assessed by comparing blank extracts from control matrices spiked at the LOQ value. The absence of peaks at the retention times of the a.i. was a criterion for confirmation method selectivity.

## 3. Results and Discussion

Honeybees may be exposed to PPPs directly or indirectly by collecting nectar and pollen. Therefore, honey can potentially contain its residues. Residue monitoring studies can reveal levels of plant protection products in honey that can vary from one substance to another. A standardized methodology for utilizing the data needed to set appropriate MRLs is unavailable. At European levels (Regulation (EU) No 283/2013, Annex 6.10 [40]), studies on residues in pollen and bee products for human consumption are necessary. Still, the type and conditions of the studies are not specified. Therefore, the MRLs for honey have historically been set at a default level of 0.05 mg kg^−1^, and a validated method should be set for residue analysis [4,28,39,40]. To date, maximum residue levels (MRLs) have not yet been established in honey and other beekeeping products; only amitraz (0.2 mg kg^−1^) and coumaphos (0.1 mg kg^−1^) have an MRL [27].

### 3.1. Multiresidue Method Validation

**Figure 1:**
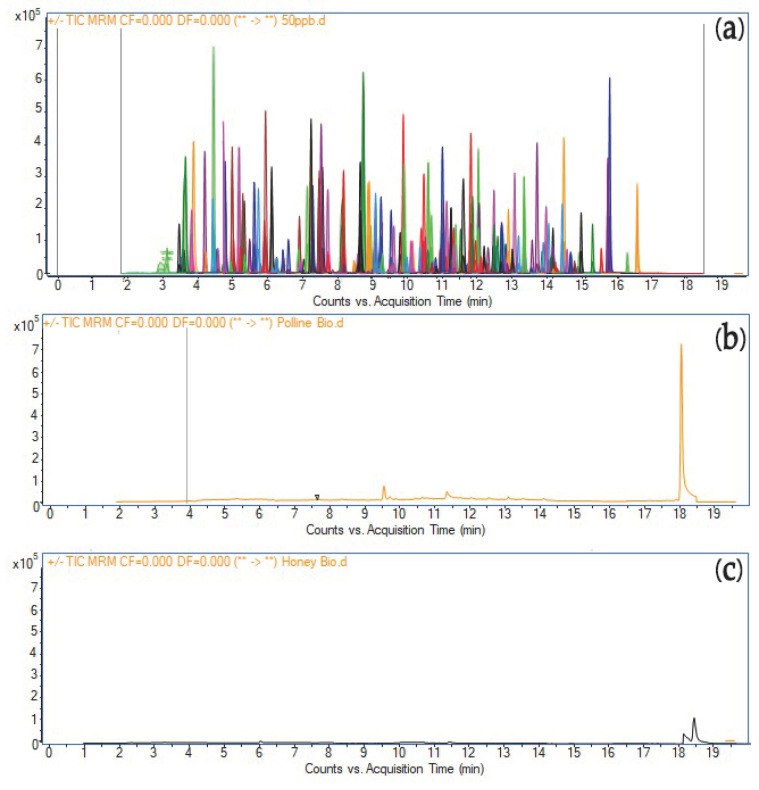
MRM chromatograms of the analytical standards (**a**), blank pollen (**b**), and blank honey (**c**).

The analysis of pesticide residues in honey samples is mainly carried out by HPLC-ESI-MS/MS and GC-MS/MS after QuEChERS sample preparation. The available methods range from 30 to 200 analytes and focus primarily on insecticides and fungicides, most of which are under revision or banned at the EU level. Kasiotis et al. (2023) analyzed 130 pesticides by HPLC-ESI-MS/MS and GC-MS/MS; 26% of the samples were positive for at least one active substance with concentrations of pesticides ranging from 1.3 ng g^−1^ to 785 ng g^−1^ in honey [41]. Those most commonly found in honey were coumaphos, imidacloprid, acetamiprid, amitraz metabolites, and tau-fluvalinate. However, minor residues of pyrethroids were also found. Pollen exhibited almost double the number of detections. Shendy et al. (2016) validated a method for 200 non-polar and polar pesticides with GC–MS/MS; the analysis of 64 honey samples showed only tau-fluvalinate in one sample [41,42,43]. The present LC-MS/MS method allowed the simultaneous determination of 237 pesticides in honey and pollen samples. No interference peaks were found in the time range of interest of the analytes, showing an acceptable selectivity of the method (Figure 1). 

The matrix effect (ME) showed that all compounds were influenced by the compounds co-extracted from the matrix. In pollen, 84% of the compounds had a suppression of the instrumental response, and 16% had an increase of the compounds co-extracted from the matrix. In contrast, the effect was reversed in the honey matrix, with 12% suppressed and 88% increased. Therefore, a quantitative analysis was performed using the calibration curves prepared in matrix extracts (Tables S1 and S2). Five-point calibration curves were prepared in honey and pollen matrix extracts in the range 0.005–0.50 mg kg^−1^; the correlation coefficients (r^2^) obtained ranged from 0.995 to 0.999 in pollen and 0.995 to 1.000 in honey, according to the SANTE Guidelines (Table S2). The method’s repeatability RSD_r_ and RSD_wr_ showed a satisfactory performance with values below 20%. The recoveries ranged from 75.3 ± 1.1% to 114.6 ± 9.0% at 0.005 mg kg^−1^ ± RSD% and from 75.5 ± 1.6% to 118.9 ± 2.8% at 0.05 mg kg^−1^ ± RSD%. According to SANTE 2021, the limit of quantification (LOQ) is defined as the lowest spike level meeting the identification and method performance criteria for recovery (70–120 %) and precision (≤20%). Therefore, the LOQ of the analytical method was set at 0.005 mg kg^−1^, far below the MRL default level of 0.05 mg kg^−1^.

### 3.2. Heavy Metals and Metalloids Analysis

The analytical methodology allowed the quantification of 27 elements among heavy metals and metalloids (Table S3). Based on the characteristics of the honey, it was necessary to preheat the honey in a water bath to obtain a homogeneous sample. When the determination could be made on more wavelengths, a quantitative analysis was performed on the most sensitive. The LOQ obtained accounted for 0.005 mg kg^−1,^ and calibration curves showed an r^2^ value ranging from 0.996 to 1.000 (Table S3). 

### 3.3. Samples Analysis

A total of 232 honey samples were collected from the different sites during the monitoring studies. The analysis of the pesticide contamination showed the absence of pesticide residues in the samples from the NW apiaries during the monitoring period. Among the apiaries from the La Maddalena Archipelago, only the sample from Caprera showed some small residues. The pesticides detected were dichlorvos (one residue), zoxamide (one residue), cycloate (three residues), and chlorantraliniprole (two residues) (Table 1). Only cycloate was found in the pollen of March and April samples; whereas, in honey samples, dichlorvos was found in November, zoxamide and chlorantraniliprole in June, and chlorantraniliprole in September. All the residues were found during 2021 monitoring except dichlorvos, which was found in 2020. Dichlorvos and chlorantraniliprole are insecticides, cycloate is an herbicide, and zoxamide is a fungicide. These pesticides are used in domestic, urban, and agricultural contexts; therefore, it is not unlikely that bees will encounter them when collecting nectar and pollen. However, the data from the monitoring studies showed an encouraging situation, with almost 95% of the samples of honey and pollen confirmed as free from pesticide residues. The literature data confirmed the low pesticide residue levels detected in honey and pollen samples, even if pollen showed a higher number of residues [41]. Pollen residue analyses in Greece, Brazil, Italy, and Egypt showed the presence of insecticides, acaricides, and fungicides, with multiresidual samples higher than single-contaminated samples. The most represented were organophosphorus, pyrethroid, and neonicotinoids among insecticides and anti-downy mildew among fungicides. Residues of azoxystrobin and mandipropamid were also found. The concentration ranged below the LOQ and MRL, and the human health risk assessment in most cases did not raise any concerns [19,24,27,41,44]. Honey samples showed similar contamination in terms of biological activity, with insecticides the most represented, followed by fungicides and herbicides. The number of positive detections was lower than that of pollen samples; multiresidual contaminations were minor, and all honey samples matched the MRLs [41,42,43,44,45,46,47]. Honey collected in organic production areas showed the absence of the investigated pesticide residues [48]. Cossu et al. (2003), after analyzing 74 honey samples collected in different areas of Sardinia, detected traces of pyrethroid pesticides in only four samples, and their concentrations were lower than the LOQ [29]. The occurrence of pesticide residues in honey results from contamination during the honeybees’ activities, such as the pursuit of food, visiting various plants, flowers, nectars, food crops, and other sources that could have been treated with agrochemicals or pesticides, and when beekeepers use chemicals to control bee pests and diseases [49,50]. This variability in the origin of the contamination can explain the high and different number of pesticides found in the literature data in honey and pollen collected in various parts of the world. According to the literature data, more than 173 pesticide residues have been encountered in apiaries worldwide [50]. According to the results reported in this study, natural vegetation honey showed fewer residues [51,52]. In 2020, the EU legislation was implemented to estimate pesticide residues in honey and to set new MRLs [28]. However, when dealing with organic honey or other apiculture products, an orientation value of 0.01 mg kg^−1^ should be taken into consideration, making it difficult to assess the level of pesticide contamination in honey and the potential harm to human health. The results found in the literature showed that the human risk assessment does not raise any concerns, and honey and pollen are unlikely to pose problems for consumer health regarding their contribution to dietary long-term exposure [50,53]. However, the literature data poses a hazard to bees’ safety and the decline of colony populations when some pesticides are employed [49]. Among the pesticides with the most significant impact on bee health, the proposed analytical method included acetamiprid, imidacloprid, dinotefuran, thiacloprid, and nitenpyram, all belonging to the family of the neonicotinoids, and fipronil (the phenylpyrazole family). No traces of these pesticides were found in the samples.

The trace and rare elements are mainly determined by inductively coupled plasma (ICP) with optical emission spectrometry (OES) or mass-spectrometry (MS); fewer papers can be found using flame atomic absorption spectroscopy (FAAS) [54]. The results are usually coupled with PCA and ANOVA statistical methods to discriminate among different floral types [55,56,57]. Only 10 elements among heavy metals and metalloids were detected in the La Maddalena and Caprera honey samples (Table 2 and Table 3). B, Ca, Fe, and Mg were represented most, followed by Zn and Al. The heavy metals Pb, Cd, As, Ni, and Hg were not detected in any sample. Ca and Mg had the higher values, accounting for 31.01 ± 11.94 and 14.86 ± 13.43 mg kg^−1^ ± RSD% in the hives of Caprera and 36.17 ± 9.81 and 13.70 ± 17.95 mg kg^−1^ ± RSD% in La Maddalena. B and Fe ranged around 3–4 mg kg^−1^, Zn ranged from 1 to 2 mg kg^−1^, and all the other elements found were below 0.5 mg kg^−1^. The ANOVA statistical analysis with Tukey correction for multiple comparisons carried out on the ICP-OES data on metals only highlighted differences for *p* < 0.05 for Ca and Mg (Table 2 and Table 3).

**Figure 2:**
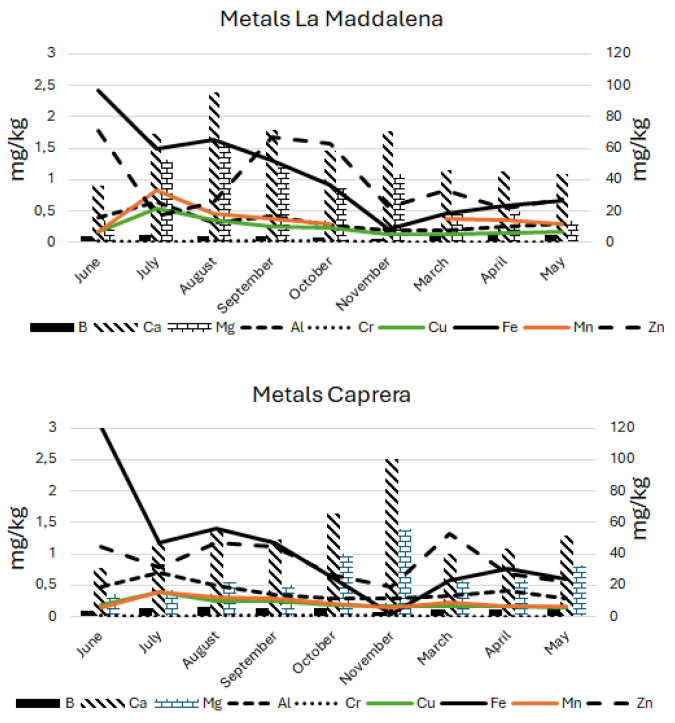
Metals quantified in honey samples from La Maddalena and Caprera.

Ca and Mg showed similar behavior in the Caprera and La Maddalena samples; however, in the La Maddalena samples, there was an increase from June to August, followed by a decrease until November, and they remained almost stable in the following period. In Caprera, there was an increase until November followed by a decrease. B, Cr, Cu, Al, and Mn showed stable values during the year. Zn showed a fluctuating trend; whereas, Fe had higher values in June, decreasing until November and then increasing again (Figure 2). 

The levels of heavy metals and metalloids in honey samples from the hive could be related to various sources, such as the degree of environmental pollution and different botanical origins. Environmental pollution can be linked to several factors, such as industry, mining, and the emission of automobile exhaust gases. Moreover, the geographical conditions are also expected to affect the mineral content. Yarsan et al. (2007) found a good correlation between the mineral content of honey and the type of flower from which bees take nectar [57]. In some cases, it was possible to distinguish the floral origin by linear discriminant analysis from their element content [58,59]. The literature data showed that, among essential elements, potassium, calcium, manganese, iron, zinc, and copper had the highest values; however, a random distribution of metal residues, showing, in some cases, differences and other similarities could be displayed [59,60,61,62]. Massidda et al. (2007) reported concentrations of lead (72.5 µg kg^−1^) and cadmium (25.5 µg kg^−1^) in samples of honey collected in a monitoring station placed near one of the most important dismantled mining areas of the southwest of Sardinia, confirming the critical role that the land plays in transmitting characteristics from the production zone to food [31]. Satta et al. (2012) studied the concentrations of cadmium, lead, and chromium in the same macro-geographical area of Sardinia. The data obtained for cadmium and lead were similar [32]. Potentially toxic elements such as arsenic, cadmium, and lead showed, in almost all cases, values close to or below the limit of quantification. Lead is the only heavy metal with a honey MRL set at 0.1 mg kg^−1^. Thus, the concentrations of toxic elements were sufficiently low to pose no risk to human health [56,57,60,63,64]. The health concerns related to metal intake in honey assessed using the Average Daily Dose (ADD), Hazard Quotients (HQs), and Hazard Index (HI) models showed that children were found to have higher estimated health risk values [64].

## 4. Conclusions

Predictive models on consuming contaminated honey and bee products showed that, when pollutants are present, even in concentrations below the prescribed MRL, serious health problems can be developed due to the accumulation of toxic substances in the body. Therefore, pesticides and heavy metals should be monitored in honey samples and bee products to protect consumers. Particular attention should be paid to samples obtained near anthropized areas and mining sites; moreover, deeper investigations should be conducted to correlate pollution to the floral origin. This paper reports two multiresidue methods for detecting contaminants in honey and pollen samples. The methods were validated and subsequently used for the analysis of original samples. In total, 237 plant protection products and 27 elements among heavy metals and metalloids were investigated in the samples collected directly from the hives in distinct locations in Sardinia. All sampling sites were characterized by a natural plant ecosystem composed of annual and perennial plant species. Anthropogenic activities, such as agricultural and industrial facilities, were far from the apiaries. The analysis showed an encouraging situation with a limited number of pesticide residues; only in the samples from Caprera, with levels close to the LOQ of the method. The random numbers of compounds in the different months and the various collecting sites were in agreement with the literature data, especially for multi-floral honey. All samples showed the absence of heavy metal contamination. Metal and metalloid contents were in the range of data found in the literature. Based on the data reported in this paper and on the literature data on organic and low-anthropized areas of honey production, no risk to human health was identified. This study confirms that honey may be a suitable biomarker of environmental contamination by pesticide residues and metals in a broad area around honeybee hives. Moreover, pollen could be used to highlight contamination from pesticides. The data reported in the literature emphasized that pollen is a much better bioindicator of heavy metals environmental contamination than honey. Therefore, future monitoring studies should focus on pollen contamination and honey to preserve children’s health and bees’ safety since a decline in the population of colonies has been detected when certain pesticides are employed.

## Figures and Tables

**Table 1:** Pesticide residue concentration (mg/kg ± RSD%) in honey and pollen samples taken in the Caprera (the La Maddalena Archipelago) hives. C_h_: honey sample; C_p_: pollen sample.

Sampling	Hives	Dichlorvos	Zoxamide	Cycloate	Chlorantraniliprole
		mg kg^−1^			
August 2020	C_h_				
November 2020	C_h_	0.008 ± 0.04			
March 2021	C_p_			0.024 ± 0.02	
April 2021	C_h_			0.029 ± 0.07	
	C_p_			0.020 ± 0.12	
June 2021	C_h_		0.031 ± 0.06		0.072 ± 0.09
September 2021	C_h_				0.007 ± 0.06

**Table 2:** Metals and metalloids (mg/kg ± RSD%) found in honey samples collected from Caprera analyzed by ICP-OES.

	Sampling Period								
	2020						2021		
Element	June	July	August	September	October	November	March	April	May
Ag	4.93 ± 15.75	2.42 ± 3.56	0.93 ± 8.31	0.57 ± 12.88	0.09 ± 16.66				
Al	0.39 ± 13.19	0.64 ± 4.02	0.32 ± 17.23	0.42 ± 12.29	0.27 ± 8.28	0.20 ± 8.86	0.20 ± 5.89	0.25 ± 15.08	0.30 ± 10.20
B	3.97 ± 10.69	1.57 ± 2.59	2.55 ± 14.42	3.58 ± 13.13	4.86 ± 2.17	3.43 ± 10.40	3.93 ± 4.50	4.60 ± 18.76	4.57 ± 11.48
Ca *	31.01 ± 11.94 ^a^	46.46 ± 13.14 ^b^	55.42 ± 15.33 ^c^	49.01 ± 6.68 ^bd^	65.65 ± 15.28 ^e^	100.16 ± 9.46 ^f^	40.40 ± 9.88 ^bg^	43.76 ± 13.53 ^bdgh^	51.91 ± 16.10 ^bcdi^
Cr	0.02 ± 17.87	<LOQ	0.03 ± 10.22	0.02 ± 15.52	0.02 ± 8.51	0.01 ± 11.98	0.01 ± 6.23	0.01 ± 12.39	0.01 ± 14.03
Cu	0.17 ± 13.42	0.54 ± 1.53	0.36 ± 14.28	0.26 ± 14.82	0.23 ± 5.42	0.12 ± 7.44	0.14 ± 12.85	0.15 ± 9.51	0.17 ± 16.90
Fe	2.43 ± 18.37	0.79 ± 8.65	0.63 ± 5.28	1.31 ± 3.10	0.91 ± 11.55	0.22 ± 9.99	0.45 ± 5.77	0.58 ± 18.28	0.66 ± 9.51
Mg *	14.86 ± 13.43 ^a^	6.76 ± 5.62 ^b^	21.94 ± 15.75 ^c^	20.22 ± 10.43 ^ac^	40.27 ± 8.58 ^d^	16.05 ± 3.69 ^ac^	23.09 ± 5.22 ^ec^	23.67 ± 15.53 ^efc^	32.81 ± 19.17 ^g^
Mn	0.18 ± 15.55	0.84 ± 4.31	0.47 ± 12.59	0.37 ± 13.18	0.30 ± 10.29	<LOQ	0.38 ± 17.66	0.35 ± 5.18	0.30 ± 17.80
Zn	0.42 ± 12.42	1.78 ± 7.05	0.65 ± 6.89	1.67 ± 7.98	1.58 ± 2.03	0.57 ± 4.12	0.83 ± 9.81	0.54 ± 6.29	0.68 ± 14.94

* different superscript letters in the row indicate statistical significance.

**Table 3:** Metals and metalloids (mg/kg ± RSD%) found in honey samples collected from La Maddalena analyzed by ICP-OES.

	Sampling Period								
	2020						2021		
Element	June	July	August	September	October	November	March	April	May
Ag	1.86 ± 1.07	0.08 ± 11.00	4.45 ± 17.29	0.12 ± 6.7	0.84 ± 10.57				
Al	0.47 ± 13.80	0.71 ± 17.03	0.50 ± 9.27	0.36 ± 13.45	0.28 ± 7.47	0.29 ± 13.70	0.34 ± 18.09	0.42 ± 18.91	0.28 ± 10.89
B	3.44 ± 19.34	5.04 ± 7.68	6.14 ± 5.98	5.76 ± 4.14	5.00 ± 5.76	2.96 ± 6.41	4.57 ± 18.29	4.45 ± 4.79	5.32 ± 9.58
Ca *	36.17 ± 9.81 ^a^	69.06 ± 15.60 ^b^	95.89 ± 8.92 ^c^	71.86 ± 14.48 ^bd^	58.76 ± 10.97 ^e^	70.85 ± 14.23 ^bdf^	45.72 ± 14.62 ^ghi^	45.33 ± 12.67 ^ghi^	43.18 ± 13.67 ^ghi^
Cr	0.02 ± 3.56	0.01 ± 8.13	0.02 ± 8.68	0.03 ± 9.43	0.02 ± 1.20	0.02 ± 1.23	0.01 ± 12.99	0.01 ± 15.43	0.01 ± 16.75
Cu	0.18 ± 13.41	0.40 ± 1.34	0.24 ± 10.58	0.24 ± 8.02	0.18 ± 11.06	0.16 ± 6.62	0.17 ± 10.52	0.16 ± 8.57	0.15 ± 6.47
Fe	3.03 ± 1.82	1.18 ± 5.35	1.41 ± 18.73	1.18 ± 9.48	0.61 ± 3.08	0.04 ± 15.70	0.58 ± 9.55	0.76 ± 17.73	0.60 ± 13.63
Mg *	13.70 ± 17.95 ^a^	52.94 ± 10.01 ^b^	61.37 ± 14.73 ^c^	48.94 ± 7.78 ^bd^	34.18 ± 13.75 ^e^	13.47 ± 15.54 ^a^	20.45 ± 13.60 ^a^	19.79 ± 8.27 ^a^	13.75 ± 10.24 ^a^
Mn	0.15 ± 14.54	0.39 ± 6.00	0.31 ± 17.03	0.28 ± 17.17	0.21 ± 3.66	0.34 ± 11.23	0.22 ± 8.21	0.17 ± 4.04	0.16 ± 12.08
Zn	1.11 ± 12.62	0.79 ± 9.29	1.17 ± 9.24	1.11 ± 17.95	0.67 ± 3.52	0.48 ± 3.91	1.33 ± 18.29	0.69 ± 7.33	0.55 ± 7.97

* different superscript letters in the row indicate statistical significance.

## Data Availability

The original contributions presented in the study are included in the article and Supplementary Material, further inquiries can be directed to the corresponding authors.

## Supplementary Materials

The following supporting information can be downloaded at: https://www.mdpi.com/article/10.3390/foods13244099/s1, Table S1: Pesticide, MRM, and validation parameters in honey and pollen matrix; Table S2: Pesticide linear regression in honey and pollen matrix, and matrix effect on pesticide analytical response; Table S3: Wavelengths, linear regression equation, coefficient of regression, and LOQ of the studied metals and metalloids.
