# Supplementary material for: Multiresidue Methods Analysis to Detect Contamination of Selected Metals in Honey and Pesticides in Honey and Pollen
Source: Foods. 2024 Dec 18;13(24):4099. doi: 10.3390/foods13244099 (PMC11675412; doi:10.3390/foods13244099)
Supplement: Supplementary file 1 [file foods-13-04099-s001.zip › foods-3377408-supplementary.pdf]

Table S1. Pesticide, MRM, and validation parameters in honey and pollen matrix.

Pesticide	Type*	MRM	LOQ mg kg^{-1}	Apparent recovery (mg kg^{-1})		RSD_r	RSD_{wR}
		ion (m/z)		0.005± RSD%	0.05± RSD%	RSD%	
Acephate	I	183.9 -> 143.0	0.005	82.8±4.9	78.2±6.1	11.9	1.5
Acetamiprid	I	223.0 -> 126.1	0.005	111.0±8.9	106.0±2.1	2.3	0.5
Alanycarb	I	400.1 -> 238.0	0.005	93.7±3.9	98.2±9.1	8.8	2.2
Aldicarb fragment	N	116.0 -> 89.1	0.005	94.5±7.4	114.9±3.3	14.7	0.9
Aldicarb	N	208.0 -> 116.0	0.005	94.8±2.4	99.9±2.0	10.2	5.8
Amidosulfuron	H	370.0 -> 261.1	0.005	76.0±2.1	118.9±2.8	10.3	4.4
Aminocarb	I	209.1 -> 137.2	0.005	88.1±3.4	82.5±1.0	7.4	2.0
Avermectin B1a	A	890.5 -> 305.1	0.005	102.4±10.7	110.3±6.1	9.8	12.5
Azaconazole	F	300.0 -> 158.9	0.005	80.8±11.1	88.2±2.8	11.4	1.2
Azamethiphos	I	325.0 -> 182.9	0.005	89.4±10.4	113.6±1.4	8.1	1.5
Azinphos-ethyl	I	346.1 -> 132.0	0.005	85.4±4.7	87.8±6.5	10.3	4.3
Azinphos-methyl	I	318.0 -> 132.1	0.005	83.0±11.5	88.4±2.2	11.0	0.7
Azoxystrobin	F	404.0 -> 372.2	0.005	97.3±12.7	112.4±3.1	7.7	0.3
Beflubutamid	H	356.0 -> 91.0	0.005	94.6±6.5	108.5±2.6	7.8	2.2
Benalaxyl	F	326.1 -> 148.1	0.005	103.4±15.0	118.6±5.7	9.5	0.6
Benfuracarb	I/N	411.1 -> 195.1	0.005	99.8±7.8	113.7±2.6	6.3	2.8
Benzoximate	A	364.1 -> 198.9	0.005	85.1±3.4	104.0±2.8	3.8	1.5
Bifenazate	A	301.1 -> 198.2	0.005	76.2±16.0	106.2±2.9	3.1	1.0
Bispyribac	H	431.0 -> 275.1	0.005	90.7±1.7	102.2±0.7	4.5	2.8
Bitertanol	F	338.1 -> 70.1	0.005	94.0±10.7	105.8±3.1	12.7	5.6
Boscalid	F	343.0 -> 307.1	0.005	93.7±3.7	102.1±0.8	14.5	2.8
Bromuconazole	F	377.9 -> 159.0	0.005	75.4±18.3	115.4±2.3	20.2	2.9
Bupirimate	F	317.1 -> 166.1	0.005	98.0±7.4	100.2±0.3	10.5	1.6
Buprofezin	I	306.1 -> 201.2	0.005	85.9±8.8	96.7±0.9	10.9	1.1
Butocarboxim	I	208.0 -> 116.0	0.005	94.8±2.4	99.9±2.0	10.2	5.8
Carbaryl	I	202.0 -> 145.0	0.005	77.6±8.1	99.8±2.8	12.2	0.6
Carbendazim	F	192.0 -> 160.1	0.005	93.2±5.6	75.5±1.6	8.8	0.8
Carbofuran	I	222.1 -> 123.1	0.005	102.5±8.2	102.2±2.8	4.0	0.3
Carbosulfan	I	381.2 -> 118.1	0.005	80.1±9.0	114.8±6.1	9.1	0.8
Carboxin	F	236.0 -> 143.0	0.005	84.1±9.6	104.5±2.3	9.3	1.0
Carfentrazone-ethyl	H	412.0 -> 366.1	0.005	108.1±4.9	94.8±2.7	10.7	1.6
Chlorantraniliprole	I	483.9 -> 452.9	0.005	89.3±10.4	101.4±6.1	1.4	4.8
Chlorfenvinphos	I	359.0 -> 155.1	0.005	101.9±13.7	81.3±1.8	16.5	1.3
Chloridazon	H	222.0 -> 77.1	0.005	91.5±11.4	84.9±2.8	7.3	0.3
Chloroxuron	H	291.0 -> 72.1	0.005	82.7±17.6	96.8±1.6	6.3	0.2
Chlorpyriphos	I	349.9 -> 197.9	0.005	101.3±3.6	97.3±0.6	11.4	0.5
Chlorpyriphos-methyl	I	322.0 -> 125.0	0.005	82.9±12.6	87.6±6.6	3.1	4.4
Chlorsulfuron	H	358.0 -> 141.1	0.005	94.2±0.3	101.2±5.0	9.6	4.5
Clofentezin	I	303.0 -> 138.0	0.005	95.5±2.0	104.8±2.3	4.2	1.5
Clomazone	H	240.0 -> 125.1	0.005	80.7±14.7	107.1±2.3	2.7	0.2
Coumaphos	I	363.0 -> 226.9	0.005	100.3±12.3	93.1±1.8	10.9	0.2
Cyazofamid	F	325.0 -> 108.1	0.005	98.7±6.2	108.5±1.3	12.9	1.6
Cycloate	H	216.1 -> 55.0	0.005	90.6±1.9	90.5±2.8	12.6	3.6
Cycluron	H	199.2 -> 72.1	0.005	97.6±5.8	99.8±3.3	9.5	0.7
Cymiazol	F	219.0 -> 144.0	0.005	78.7±6.2	82.3±2.1	4.9	1.5
Cymoxanil	F	199.0 -> 128.1	0.005	88.5±9.1	105.5±2.7	12.9	1.6
Cyproconazole	F	292.1 -> 70.1	0.005	75.8±6.7	89.2±3.3	13.4	1.7
Cyprodinil	F	226.1 -> 93.1	0.005	96.9±14.9	97.2±3.1	5.8	1.6
DEET	I	192.1 -> 119.0	0.005	82.8±8.1	99.8±2.5	3.6	1.6
Desmedipham	H	318.1 -> 182.2	0.005	100.8±1.7	106.5±2.0	12.9	5.7
Dichlorvos	I	305.1 -> 169.0	0.005	103.4±5.8	100.5±7.7	11.1	1.2
Diethofencarb	F	221.0 -> 109.0	0.005	89.7±13.0	105.3±0.8	8.1	0.6
Difenoconazole	F	268.1 -> 124.0	0.005	87.1±16.1	94.8±5.1	7.4	0.8
Diflubenzuron	I	406.0 -> 337.1	0.005	96.9±4.8	97.9±4.5	12.2	1.0
Diflufenican	H	311.0 -> 158.0	0.005	88.5±10.0	93.8±1.6	12.7	2.4
Dimethoate	I	230.0 -> 125.0	0.005	88.0±9.4	107.8±1.1	5.9	1.4
Dimethomorph	F	388.0 -> 301.1	0.005	94.0±11.4	104.3±1.6	4.6	3.5

Dimoxystrobin	F	327.1 -> 205.1	0.005	81.6±9.0	89.6±1.2	5.8	1.1
Diniconazole	F	326.0 -> 70.1	0.005	90.3±8.6	101.5±2.1	10.9	1.6
Dinotefuran	I	203.1 -> 129.0	0.005	114.6±9.0	99.0±12.2	3.9	9.3
Dioxacarb	I	224.0 -> 167.0	0.005	85.4±9.9	100.7±0.5	9.1	1.5
Disulfoton	I	275.0 -> 89.0	0.005	97.2±4.4	78.5±7.3	13.8	15.7
Diuron	H	233.0 -> 72.1	0.005	84.5±7.4	106.6±2.3	7.1	0.9
Epoxyconazole	F	330.0 -> 121.1	0.005	89.4±5.3	118.7±0.5	3.9	1.9
Ethidimuron	H	265.1 -> 207.9	0.005	76.2±19.3	111.0±1.9	4.4	0.2
Ethion	I	385.0 -> 199.0	0.005	106.9±8.6	106.5±2.8	14.5	1.1
Ethirimol	F	210.1 -> 140.1	0.005	101.0±4.7	102.1±3.9	9.9	0.0
Ethofumesat	H	287.0 -> 121.1	0.005	102.9±4.8	91.0±5.6	16.0	11.5
Ethoprophos	I	243.0 -> 97.0	0.005	93.3±15.6	103.6±0.9	9.7	1.1
Ethoxyquin	H	218.0 -> 174.0	0.005	93.5±2.3	99.5±2.3	4.3	3.1
Etofenprox	I	394.2 -> 177.2	0.005	95.3±6.9	108.5±0.5	3.3	0.5
Famoxadone	F	392.1 -> 331.2	0.005	108.4±5.7	106.8±3.5	15.0	6.4
Fenamidone	F	312.1 -> 92.1	0.005	92.3±8.8	112.3±2.5	12.6	1.2
Fenamiphos	I	304.1 -> 217.1	0.005	94.3±8.3	91.5±3.6	8.9	2.6
Fenazaquin	A	307.2 -> 57.1	0.005	89.7±5.0	94.0±2.2	17.5	1.5
Fenbuconazole	F	337.1 -> 125.1	0.005	105.2±1.6	101.5±6.3	11.8	3.6
Fenhexamid	F	302.0 -> 97.2	0.005	89.0±5.5	112.6±8.1	12.1	3.1
Fenobucarb	I	208.1 -> 95.1	0.005	91.2±7.1	115.0±3.7	16.2	1.5
Fenoxycarb	I	302.1 -> 88.1	0.005	75.8±15.2	107.4±2.5	8.7	1.0
Fenpropidin	F	274.0 -> 147.0	0.005	88.7±7.5	86.8±6.5	11.2	9.6
Fenpyroximat	I	422.1 -> 366.2	0.005	96.9±5.3	113.9±1.7	17.7	0.9
Fenuron	H	165.1 -> 72.0	0.005	81.6±9.2	101.4±2.4	10.2	0.8
Fipronil	I	435.0 -> 330.0	0.005	95.7±7.9	98.7±5.4	9.0	3.9
Flazasulfuron	H	407.9 -> 182.1	0.005	77.7±1.0	84.9±12.9	16.4	2.1
Flonicamid	I	230.0 -> 203.0	0.005	88.3±11.4	98.8±3.4	9.4	0.8
Fluazinam	F	462.9 -> 415.9	0.005	81.0±6.6	92.7±1.9	3.1	3.6
Flubendiamide	I	681.0 -> 254.0	0.005	105.0±16.2	98.5±6.9	7.7	5.2
Flufenacet	H	364.0 -> 194.2	0.005	81.2±5.6	97.0±1.6	6.0	2.0
Flufenoxuron	I	489.0 -> 158.0	0.005	88.4±13.7	105.8±1.9	5.3	0.5
Flumetsulam	H	326.0 -> 129.2	0.005	98.9±0.7	91.7±3.7	17.1	1.2
Flumioxazin	H	355.1 -> 327.1	0.005	76.4±14.7	109.1±4.3	11.8	15.9
Fluometuron	H	233.1 -> 72.1	0.005	90.7±11.1	106.9±1.1	9.8	1.2
Fluopicolide	F	382.9 -> 172.9	0.005	76.1±18.5	92.3±4.9	10.2	2.6
Fluoxastrobin	F	459.0 -> 427.1	0.005	112.3±12.7	109.8±3.3	9.3	1.1
Fluquinconazole	F	376.0 -> 307.0	0.005	101.4±2.2	98.9±13.0	10	3.0
Flusilazole	F	316.0 -> 247.2	0.005	85.8±18.3	95.4±1.0	13.0	0.8
Flutriafol	F	302.0 -> 70.1	0.005	87.5±4.0	101.3±0.9	11.0	0.7
Foramsulfuron	H	453.1 -> 182.1	0.005	75.3±1.1	101.0±1.4	10.0	5.4
Fosthiazate	N	284.0 -> 104.1	0.005	76.0±6.5	86.2±1.9	10.0	1.4
Fuberidazol	F	185.1 -> 157.1	0.005	75.9±4.3	106.0±2.1	6.5	0.4
Furalaxyl	F	302.1 -> 95.0	0.005	85.2±9.1	102.3±1.3	8.2	0.6
Furathiocarb	I	383.1 -> 195.1	0.005	94.1±5.1	101.1±3.7	6.9	1.3
Halofenozide	I	329.1 -> 120.9	0.005	82.0±2.9	116.2±2.1	6.1	3.8
Hexaconazole	F	314.0 -> 70.1	0.005	77.4±2.1	100.4±2.9	3.3	3.0
Hexaflumuron	I	461.0 -> 158.0	0.005	99.3±1.3	107.8±1.6	6.5	0.9
Hexythiazox	I	353.0 -> 228.1	0.005	93.1±5.1	89.5±1.7	11.0	1.7
Hydramethylnon	I	495.2 -> 323.0	0.005	76.3±7.3	88.1±3.2	4.6	1.2
Imazalil	F	297.0 -> 159.0	0.005	88.1±2.9	101.6±3.4	5.4	2.1
Imidacloprid	I	256.0 -> 175.1	0.005	78.0±3.2	112.5±2.0	3.7	0.9
Indoxacarb	I	528.0 -> 203.0	0.005	102.7±11.6	99.7±3.5	6.3	4.5
Ipconazole	F	334.1 -> 70.0	0.005	93.7±17.2	116.8±3.4	12.5	13.1
Iprovalicarb	F	321.2 -> 119.1	0.005	99.4±9.2	108.0±3.2	7.7	2.5
Isocarbophos	I	231.0 -> 121.0	0.005	85.0±16.5	102.0±0.4	8.2	0.8
Isofenphos-methyl	I	332.0 -> 121.0	0.005	93.3±6.3	93.1±3.0	11.3	3.4
Isoprothiolane	F	291.1 -> 231.0	0.005	77.9±8.0	107.6±1.4	8.8	2.1
Isoxaben	H	333.2 -> 165.0	0.005	85.5±10.4	99.9±0.1	10.6	0.4
Ivermectin B1a	N	892.5 -> 307.2	0.005	103.4±15.0	89.6±6.2	6.2	8.6
Kresoxim-methyl	F	314.1 -> 267.1	0.005	106.7±11.1	100.1±1.7	4.9	4.0
Lenacil	H	235.2 -> 153.1	0.005	106.8±4.5	97.6±2.6	6.8	1.2

Linuron	H	249.0 -> 160.0	0.005	80.9±5.4	103.6±1.6	15.8	0.5
Lufenuron	I	510.9 -> 158.0	0.005	91.9±6.2	103.1±4.1	8.6	4.0
Malaoxon	I	315.1 -> 99.0	0.005	96.0±5.3	107.6±1.2	8.6	0.6
Malathion	I	331.0 -> 126.9	0.005	112.4±16.3	92.9±2.5	13.4	6.7
Mandipropamid	F	411.9 -> 328.1	0.005	96.7±8.0	107.1±3.5	7.8	2.0
Mecarbam	I/A	330.0 -> 97.1	0.005	99.8±9.0	110.3±2.9	6.8	0.5
Mepanipyrim	F	224.0 -> 106.1	0.005	76.3±8.6	100.9±3.7	4.0	1.0
Mesosulfuron-methyl		504.1 -> 182.1	0.005	82.5±1.1	97.8±3.8	12.5	14.3
Metaflumizone	I	507.0 -> 178.1	0.005	97.3±6.3	110.1±4.9	18.6	6.9
Metalaxyl	F	280.1 -> 220.2	0.005	88.8±7.0	106.2±2.1	8.1	0.4
Metamitron	H	203.1 -> 175.1	0.005	93.6±13.1	87.7±2.4	0.4	0.9
Metazachlor	H	278.0 -> 134.1	0.005	83.1±13.6	106.4±1.6	12.4	0.1
Metconazole	H	320.1 -> 70.1	0.005	78.2±5.1	98.4±2.1	11.6	1.2
Methabenzthiazuron	F	222.0 -> 165.0	0.005	102.0±8.1	97.5±3.0	5.6	0.4
Methacrifos	H	241.0 -> 125.1	0.005	86.4±12.3	81.1±2.5	0.7	0.9
Methamidophos	I	141.9 -> 94.1	0.005	95.8±8.9	78.9±2.6	14.8	2.2
Methidathion	I	302.9 -> 85.1	0.005	78.5±15.3	76.2±2.1	14.8	1.3
Methiocarb	I	226.1 -> 121.1	0.005	85.3±9.0	99.0±2.0	13.3	2.6
Methomyl	I	162.9 -> 106.1	0.005	104.2±19.5	101.8±2.4	9.5	1.0
Methoprotryne	H	272.2 -> 198.0	0.005	80.6±6.1	97.9±2.5	7.8	1.6
Methoxyfenozide	I	369.2 -> 149.1	0.005	96.7±3.6	112.0±3.0	5.7	4.2
Metobromuron	H	259.0 -> 170.0	0.005	90.1±7.7	108.4±4.1	11.1	0.2
Metolachlor	H	284.1 -> 252.2	0.005	90.4±7.7	105.4±2.7	7.0	0.8
Metrafenon	F	409.0 -> 209.1	0.005	92.6±6.5	111.1±1.6	9.6	1.8
Metsulfuron-methyl	H	382.0 -> 167.1	0.005	75.5±0.5	99.6±0.3	5.4	2.2
Mevinphos	I	225.0 -> 127.0	0.005	90.3±12.2	106.3±1.0	4.8	1.2
Mexacarbate	I	223.1 -> 151.0	0.005	77.1±10.0	100.8±1.9	7.6	1.0
Molinate	A	188.1 -> 126.0	0.005	76.5±4.4	106.5±5.1	9.8	1.4
Monocrotophos	I	224.0 -> 127.0	0.005	84.2±9.7	95.5±1.4	13.0	1.0
Moxidectin	I	640.4 -> 622.2	0.005	113.2±10.0	96.6±4.5	8.4	1.9
Myclobutanil	F	289.1 -> 70.1	0.005	83.6±6.8	93.2±2.1	1.1	1.9
Nicosulfuron	H	411.1 -> 182.1	0.005	99.3±0.8	104.0±7.5	4.5	5.2
Nitenpyram	I	271.1 -> 56.1	0.005	79.4±4.4	90.2±3.9	1.8	1.2
Novaluron	I	493.1 -> 158.1	0.005	96.0±10.1	113.4±3.5	6.8	3.2
Omethoate	I	214.0 -> 125.0	0.005	83.7±5.6	93.3±1.4	10.1	0.7
Oxadiazon	H	345.0 -> 303.0	0.005	100.3±12.0	108.9±1.9	15.8	1.2
Oxamyl	I	237.0 -> 72.1	0.005	89.0±6.4	103.4±2.1	6.7	0.4
Oxasulfuron	H	407.0 -> 150.1	0.005	99.9±0.1	104.8±2.6	3.1	1.4
Paclobutrazol	F	294.1 -> 70.1	0.005	90.0±19.2	104.6±3.7	11.2	1.3
Penconazole	F	284.0 -> 70.1	0.005	80.9±6.8	102.8±2.8	7.4	1.9
Pencycuron	F	329.1 -> 125.0	0.005	81.2±10.7	102.2±1.8	6.1	1.1
Pendimethalin	H	282.1 -> 212.1	0.005	90.0±10.9	110.0±2.5	6.4	1.1
Phenmedipham	H	318.1 -> 136.0	0.005	98.6±1.7	101.5±1.2	7.3	2.8
Phenthoate	I	321.0 -> 79.1	0.005	96.2±6.6	113.4±1.1	9.8	1.0
Phosalone	I	368.0 -> 182.0	0.005	102.6±7.8	96.0±1.2	17.6	4.1
Phosmet	I	317.9 -> 160.0	0.005	81.6±8.7	92.7±2.1	16.0	2.4
Phosphamidone	I	300.1 -> 174.1	0.005	84.4±8.8	106.5±2.2	6.2	0.4
Phoxim	I	299.1 -> 77.1	0.005	91.0±9.9	106.7±0.4	6.7	2.1
Picolinafen	H	377.1 -> 238.0	0.005	93.4±8.5	108.6±1.8	11.3	2.1
Picoxystrobin	F	368.1 -> 145.1	0.005	105.2±8.3	95.1±1.8	4.0	1.1
Pirimicarb	I	239.1 -> 72.1	0.005	88.1±5.8	104.4±2.3	5.5	1.1
Pirimiphos-methyl	I	306.0 -> 164.2	0.005	97.8±6.9	110.6±1.8	9.7	1.5
Prochloraz	F	376.0 -> 308.0	0.005	86.8±12.1	84.5±2.6	6.4	3.2
Profenofos	I	374.9 -> 304.9	0.005	88.0±7.9	105.9±1.6	9.0	2.1
Promecarb	I	208.1 -> 109.1	0.005	86.7±8.4	102.9±0.9	9.1	2.2
Prometon	H	226.2 -> 142.1	0.005	76.0±2.9	92.3±1.6	5.6	0.2
Propamocarb	F	189.1 -> 102.1	0.005	75.6±0.7	96.2±1.0	8.1	0.8
Propaquizafop	H	444.0 -> 100.2	0.005	93.3±3.2	104.7±0.8	8.6	1.8
Propargite	I	368.1 -> 231.2	0.005	94.1±8.2	106.8±2.9	15.5	0.2
Propetamophos	I	282.1 -> 138.0	0.005	101.3±17.1	112.1±4.9	6.6	3.0
Propham	H	180.1 -> 138.1	0.005	87.8±8.1	109.9±1.0	13.6	1.6
Propiconazole	F	342.0 -> 158.9	0.005	100.6±1.5	118.6±5.7	3.5	2.6

Propoxur	I	210.1 -> 111.1	0.005	91.2±5.1	101.5±1.8	9.3	1.0
Propyzamid	H	256.0 -> 173.0	0.005	82.2±6.4	102.8±2.4	13.6	0.5
Proquinazid	F	372.9 -> 289.0	0.005	102.4±9.1	89.3±1.6	5.7	0.8
Prosulfocarb	H	252.1 -> 91.1	0.005	89.8±6.1	105.7±2.8	8.4	1.0
Pymetrozine	I	218.0 -> 105.1	0.005	100.9±2.9	83.0±1.6	3.9	0.4
Pyracarbolid	F	218.1 -> 125.0	0.005	75.8±10.5	88.0±1.9	8.6	0.3
Pyraclostrobin	F	388.0 -> 163.1	0.005	92.1±11.3	88.3±1.6	2.8	2.1
Pyridaben	A	365.1 -> 147.1	0.005	88.4±11.0	109.3±2.3	13.6	0.9
Pyridat	H	378.9 -> 350.8	0.005	99.1±0.6	97.1±2.7	5.4	14.4
Pyrimethanil	F	200.1 -> 82.1	0.005	92.9±17.9	94.4±1.5	5.2	1.1
Pyriproxyfen	I	322.1 -> 96.1	0.005	97.6±6.8	113.1±2.3	13.2	0.6
Quinalphos	I	299.0 -> 163.0	0.005	89.0±6.0	109.4±0.9	5.2	3.5
Quinmerac	H	222.0 -> 204.0	0.005	99.4±0.1	100.0±0.1	13.0	1.5
Quinoclamin	H	208.0 -> 76.9	0.005	76.4±14.0	90.8±3.6	8.7	1.5
Quinoxyfen	F	308.0 -> 197.0	0.005	79.3±4.8	97.7±3.5	18.7	0.7
Rimsulfuron	H	432.1 -> 182.1	0.005	90.8±0.2	95.8±3.4	10.9	19.4
Rotenone	I	395.0 -> 213.1	0.005	96.1±3.3	115.7±6.4	2.7	1.4
Secbumeton	H	226.2 -> 170.1	0.005	76.0±1.3	88.3±1.6	5.3	0.7
Silthiopham	F	268.0 -> 252.1	0.005	87.3±9.0	105.4±3.0	3.1	1.5
Spinosyn A	I	732.4 -> 142.1	0.005	76.9±5.0	94.2±1.8	2.8	1.4
Spinosyn D	I	746.5 -> 142.1	0.005	82.0±7.2	99.8±3.2	2.8	1.9
Spirodiclofen	I	411.1 -> 71.2	0.005	96.0±0.9	93.4±1.0	5.5	5.5
Spiromesifen	I	388.2 -> 273.0	0.005	93.2±4.8	108.4±1.4	10.2	9.7
Spirotetramat	I	374.1 -> 330.3	0.005	81.6±10.0	83.1±1.9	13.6	4.3
Sulfentrazone	H	404.0 -> 273.0	0.005	92.6±5.7	77.2±15.6	4.2	4.7
Tebuconazole	F	308.1 -> 70.1	0.005	78.1±12.0	81.4±2.2	1.0	0.9
Tebufenozid	H	353.0 -> 133.1	0.005	97.2±7.3	85.9±3.2	2.6	2.7
Tebufenpyrad	I	334.1 -> 117.1	0.005	109.0±5.6	89.5±1.3	9.1	1.6
Tebuthiuron	I	229.1 -> 172.1	0.005	104.0±10.2	95.8±1.7	10.2	1.0
Teflubenzuron	H	379.0 -> 339.0	0.005	112.9±1.8	81.3±2.4	14.8	1.2
Tepraloxydim	H	342.1 -> 250.2	0.005	75.7±6.6	77.3±15.5	8.1	8.1
Terbufos	I	289.1 -> 57.1	0.005	88.4±2.1	82.3±1.4	11.1	3.0
Tetraconazole	F	372.0 -> 159.0	0.005	82.3±12.0	77.1±1.6	8.9	1.7
Thiabendazole	F	202.0 -> 175.1	0.005	77.5±2.7	78.7±9.0	8.9	0.6
Thiacloprid	I	253.0 -> 126.0	0.005	92.5±6.9	90.2±1.6	2.9	0.5
Thifensulfuron-methyl	H	388.0 -> 167.1	0.005	79.8±0.4	94.6±2.3	8.0	1.2
Thiodicarb	I	355.0 -> 88.1	0.005	79.1±8.3	101.9±1.4	7.4	0.7
Thiofanox	I	241.1 -> 184.1	0.005	79.1±3.4	109.7±5.3	6.9	1.3
Tolclofos-methyl	F	300.9 -> 125.0	0.005	87.3±0.8	108.6±1.5	7.9	3.2
Tolylfluanide	F	346.9 -> 137.0	0.005	92.1±2.8	89.7±9.9	6.7	4.2
Tralkoxydim	H	330.1 -> 138.1	0.005	95.6±2.0	99.8±1.6	16.2	0.8
Triadimefon	F	294.1 -> 69.1	0.005	92.6±12.5	112.2±1.9	13.1	1.6
Triadimenol	F	296.1 -> 70.1	0.005	104.9±15.0	91.5±1.9	13.8	4.6
Triasulfuron	H	401.9 -> 167.1	0.005	75.6±0.7	97.6±6.2	15.2	5.5
Triazophos	I	314.0 -> 162.1	0.005	86.5±17.9	111.8±4.7	8.3	1.6
Trichlorfon	I	256.9 -> 221.0	0.005	93.6±3.4	85.8±2.9	8.0	0.9
Tricyclazol	F	190.0 -> 136.0	0.005	76.5±5.8	84.7±3.9	5.0	0.5
Trietazin	H	230.1 -> 99.0	0.005	92.4±10.2	102.8±2.2	7.9	1.6
Trifloxystrobin	F	409.1 -> 186.1	0.005	104.3±12.5	116.6±2.6	13.3	0.8
Triflumizol	F	346.0 -> 73.1	0.005	81.6±11.1	106.4±3.7	6.9	1.2
Triflumuron	I	359.0 -> 156.0	0.005	92.9±7.7	105.2±1.0	9.1	1.4
Trimethacarb	I	194.1 -> 137.0	0.005	83.9±6.4	101.0±2.3	14.8	0.8
Triticonazole	F	318.1 -> 70.1	0.005	78.0±9.0	114.9±8.9	0.9	3.8
Uniconazole-P	H	292.1 -> 70.0	0.005	87.0±4.8	87.6±2.4	7.8	0.8
Vamidothion	I	288.1 -> 146.0	0.005	93.2±4.9	115.9±2.4	8.0	0.6
Zoxamide	F	336.0 -> 187.0	0.005	89.3±5.2	105.1±3.0	6.8	0.8

* I: Insecticide; F: Fungicide; H: herbicide; A: Acaricide; N: Nematocide;

Table S2. Pesticide linear regression in honey and pollen matrix, and matrix effect on pesticide analytical response.

Pesticide	Linear regression equation in Honey	r² ± RSD%	Linear regression equation in pollen	r² ± RSD%	ME% honey	ME% pollen
Acephate	Y=4410.13X+6225.94	0.999±0.03	Y=8703.58X-6354.69	0.999±0.01	-1.6	-6.4
Acetamiprid	Y=27437.84X-225972.26	0.995±0.64	Y=11821.88X-133884.15	0.996±0.42	17.4	-17.0
Alanycarb	Y=6999.84X-17850.62	0.996±0.97	Y=1845.13X+5744.88	0.995±0.70	-90.5	-24.6
Aldicarb fragment	Y=9014.17X-19517.78	0.996±0.25	Y=2993.49X+151.84	0.999±0.16	16.2	-20.1
Aldicarb	Y=16008.37X-27547.10	0.994±0.38	Y=6877.49X-2120.86	0.996±0.43	19.3	-15.1
Amidosulfuron	Y=11293.78X-34827.82	0.991±0.58	Y=2047.36X-8752.75	0.997±0.30	31.2	43.0
Aminocarb	Y=100415.5X+599823.56	0.998±0.10	Y=23156.65X-139248.44	0.999±0.06	-12.4	-53.0
Avermectin B1a	Y=33.39X-55.51	1.000±0.00	Y=11.89X+92.41	0.997±0.23	1.1	-5.0
Azaconazole	Y=18646.26X-113197.62	0.995±0.37	Y=6331.81X-27357.62	0.997±0.37	20.6	-2.4
Azamethiphos	Y=37705.24X-70222.70	0.997±0.17	Y=10040.74X-20631.03	0.999±0.06	16.1	-26.1
Azinphos-ethyl	Y=4283.86X-38793.55	0.996±0.63	Y=1497.92X-7400.39	0.995±0.73	43.2	11.3
Azinphos-methyl	Y=6253.02X-66311.32	0.996±1.55	Y=1899.35X-4835.20	0.995±0.90	98.4	1.0
Azoxystrobin	Y=21564.21X-31575.01	0.996±0.40	Y=21564.21X-31575.01	0.997±0.39	23.6	-22.5
Beflubutamid	Y=31609.54X-293741.33	0.996±1.12	Y=11783.53X-92230.28	0.995±0.91	18.9	-15.3
Benalaxyl	Y=51274.23X-233032.31	0.995±0.31	Y=13293.89X-46448.53	0.998±0.26	25.1	-26.8
Benfuracarb	Y=12629.62X-5035.24	0.995±0.48	Y=1109.47X+3591.31	0.995±1.01	28.4	-15.6
Benzoximate	Y=37211.03X-83035.03	0.999±0.06	Y=11529.60X-17171.96	0.999±0.10	20.4	-17.8
Bifenazate	Y=35580.60X-274959.77	0.997±0.44	Y=12045.67X-37683.53	0.996±0.62	37.7	-10.9
Bispyribac	Y=4697.83X-5163.02	0.998±0.15	Y=1245.90X+5583.55	0.999±0.09	17.0	-11.2
Bitertanol	Y=1735.96X+5782.30	0.998±0.10	Y=349.81X+2571.56	0.998±0.11	-8.5	-16.5
Boscalid	Y=4021.71X-10385.51	0.998±0.08	Y=1130.26X-801.65	0.999±0.08	4.0	-12.5
Bromuconazole	Y=2464.04X-2973.67	0.999±0.03	Y=632.04X+558.92	0.999±0.06	10.0	-22.7
Bupirimate	Y=18786.23X-99282.35	0.995±0.47	Y=4664.04X-3987.24	0.997±0.50	33.6	-33.0
Buprofezin	Y=44785.62X-108163.48	0.997±0.19	Y=12320.04X-15559.14	0.999±0.15	23.1	-15.1
Butocarboxim	Y=16008.37X-27547.10	0.995±0.38	Y=6877.49X-2120.86	0.996±0.43	19.3	-15.1
Carbaryl	Y=10265.61X-28790.56	0.998±0.05	Y=4147.82X-13558.50	0.999±0.04	8.7	-18.3
Carbendazim	Y=98452.13X-1604.54	0.998±0.11	Y=41642.66X-31084.66	0.999±0.00	-11.2	-16.7
Carbofuran	Y=51636.82X-191915.75	0.996±0.37	Y=18588.17X-21695.79	0.998±0.33	-23.3	-45.9
Carbosulfan	Y=38513.05X-34952.99	0.999±0.09	Y=4878.04X-41.28	0.998±0.15	34.0	17.6
Carboxin	Y=57793.13X-123608.42	0.996±0.22	Y=17460.93X-41711.63	0.999±0.14	60.3	-16.9
Carfentrazone-ethyl	Y=6133.86X-34938.99	0.995±0.21	Y=1813.63X-8258.19	0.997±0.42	18.1	-10.3
Chlorantraniliprole	Y=3574.27X-20883.36	0.995±0.27	Y=1021.95X-1600.43	0.997±0.42	7.3	-17.4
Chlorfenvinphos	Y=10833.77X-77247.59	0.996±0.68	Y=3261.55X-16382.38	0.996±0.42	34.9	-28.1
Chloridazon	Y=13005.20X-12894.14	0.997±0.20	Y=6574.58X-14389.34	0.999±0.09	3.5	-15.2
Chloroxuron	Y=40895.16X-265288.35	0.995±0.42	Y=14623.95X-66582.75	0.997±0.35	17.5	-11.4
Chlorpyriphos	Y=5596.55X-10060.71	0.999±0.03	Y=1561.83X-282.21	0.999±0.04	12.3	-30.4
Chlorpyriphos-methyl	Y=2049.44X+1403.36	1.000±0.02	Y=534.04X+478.87	0.999±0.05	15.9	43.3
Chlorsulfuron	Y=4143.77X+2830.82	1.000±0.02	Y=781.91X+3499.67	0.999±0.10	1.6	-22.2
Clofentezin	Y=14967.24X-81453.16	0.995±0.32	Y=4877.91X-5774.22	0.997±0.45	13.6	15.2
Clomazone	Y=33547.87X-154153.75	0.995±0.42	Y=12149.26X-48719.29	0.998±0.16	10.3	-17.1
Coumaphos	Y=14744.24X-108820.25	0.996±0.26	Y=4072.25X-16594.05	0.997±0.39	27.7	-34.9
Cyazofamid	Y=30071.22X-73073.64	0.997±0.50	Y=8883.02X-27641.09	0.998±0.25	57.9	-21.3
Cycloate	Y=6418.78X-13603.91	0.997±0.19	Y=2213.83X-1059.61	0.998±0.14	33.6	1.6
Cycluron	Y=43818.11X-231347.97	0.995±0.39	Y=15977.58X-58301.80	0.997±0.42	14.9	-12.2
Cymiazol	Y=14328.81X+11030.98	1.000±0.03	Y=5585.18X+6621.06	0.999±0.01	-9.7	-6.7
Cymoxanil	Y=6016.90X+169.07	0.999±0.07	Y=2933.36X-813.22	0.999±0.01	2.1	-11.6
Cyproconazole	Y=12385.33X-34139.57	0.997±0.15	Y=3131.11X-23253.51	0.999±0.04	22.4	43.1
Cyprodinil	Y=21266.68X-1638.12	0.999±0.05	Y=6500.59X+1831.69	0.999±0.04	-6.5	59.0
DEET	Y=112940.84X-354390.25	0.995±0.43	Y=41847.62X-73404.22	0.997±0.40	17.1	-15.2
Desmedipham	Y=23453.69X-56255.50	0.998±0.10	Y=6299.98X+19623.65	0.997±0.23	-3.1	-19.1
Dichlorvos	Y=4573.24X+1054.19	1.000±0.02	Y=1930.04X-3003.19	0.998±0.21	13.1	-14.6
Diethofencarb	Y=26053.96X-132787.89	0.996±0.31	Y=8382.74X-39688.32	0.999±0.01	33.3	-7.6
Difenoconazole	Y=7012.88X-3838.52	1.000±0.03	Y=1607.47X-892.71	0.997±0.26	6.2	50.8
Diflubenzuron	Y=9939.18X-19422.10	0.995±0.35	Y=2928.47X-14250.61	0.999±0.01	3.0	-14.3
Diflufenican	Y=15928.46X-74748.54	0.996±0.14	Y=4659.40X-15622.97	0.998±0.16	11.4	-10.2
Dimethoate	Y=22557.15X-132850.38	0.995±0.52	Y=12495.15X-47911.20	0.999±0.12	-6.6	-15.4

Dimethomorph	Y=10025.70X-15499.50	0.999±0.06	Y=2478.54X-2347.49	0.997±0.31	10.7	13.7
Dimoxystrobin	Y=90240.10X-247772.53	0.995±0.32	Y=25483.11X-38345.94	0.999±0.04	26.8	-9.2
Diniconazole	Y=6580.70X-18976.75	0.997±0.15	Y=1485.63X+1266.82	0.996±0.51	21.6	-25.6
Dinotefuran	Y=24.27X-62.58	0.996±1.39	Y=56.86X-211.71	0.999±0.07	-29.3	-69.9
Dioxacarb	Y=24481.86X-110806.04	0.997±0.04	Y=12259.78X-47790.66	0.996±0.46	429.4	71.9
Disulfoton	Y=48.67X-244.72	0.995±0.20	Y=7.78X+8.83	0.999±0.02	7.1	-29.1
Diuron	Y=51599.81X-331565.65	0.996±0.38	Y=20752.11X-68200.66	0.999±0.78	0.0	-9.4
Epoxyconazole	Y=23774.96X-13749.02	1.000±0.03	Y=5054.96X+819.85	0.997±0.29	15.1	-27.9
Ethidimuron	Y=19108.52X-45768.33	0.998±0.06	Y=4987.54X-25323.02	0.999±0.01	9.9	-31.4
Ethion	Y=35542.29X-104288.55	0.998±0.09	Y=9549.10X-4272.27	0.999±0.08	19.3	-31.4
Ethirimol	Y=31246.05X+21105.15	1.000±0.03	Y=11354.88X-15256.86	0.999±0.15	-14.4	-12.2
Ethofumesat	Y=388.40X-1276.83	0.995±0.31	Y=73.46X-523.79	0.999±0.07	22.4	6.9
Ethoprophos	Y=30373.04X-77733.89	0.997±0.18	Y=7886.24X-19367.95	0.997±0.25	23.9	-29.6
Ethoxyquin	Y=7633.57X-7670.66	0.995±0.34	Y=2689.91X-30474.83	0.999±0.08	28.8	-15.6
Etofenprox	Y=56550.06X-379537.05	0.997±0.21	Y=14835.01X-41498.38	0.996±7.11	13.5	-18.7
Famoxadone	Y=1310.29X-5681.19	0.995±0.26	Y=370.24X-23.80	0.996±0.58	8.2	-16.7
Fenamidone	Y=23105.82X-77401.72	0.998±0.05	Y=6651.17X-15447.43	0.999±0.11	-2.6	-18.6
Fenamiphos	Y=52366.95X-265620.39	0.995±0.40	Y=14952.03X-66574.60	0.999±0.03	28.8	-2.6
Fenazaquin	Y=114809.33X-325278.78	0.997±0.87	Y=32788.64X-8759.88	0.997±0.42	29.5	-24.1
Fenbuconazole	Y=7824.48X-9944.63	0.999±0.05	Y=1694.88X-2398.71	0.995±0.91	17.1	-24.4
Fenhexamid	Y=5355.70X-25446.30	0.996±0.38	Y=1411.78X-5176.40	0.998±0.22	20.8	-10.1
Fenobucarb	Y=21861.44X-4609.21	1.000±0.03	Y=8113.90X-5906.49	0.998±0.25	1.2	-23.5
Fenoxycarb	Y=22323.86X-146145.42	0.997±0.49	Y=7065.35X-26502.87	0.999±0.01	29.4	-10.8
Fenpropidin	Y=37795.21X-76628.45	0.998±0.10	Y=66.42X-386.33	0.997±0.36	8.0	-20.9
Fenpyroximat	Y=37277.57X+15465.48	1.000±0.02	Y=8020.18X-9567.56	0.999±0.02	10.9	-27.8
Fenuron	Y=1828.67X-6970.18	0.995±0.32	Y=922.89X-147.87	0.996±0.54	7.5	-11.7
Fipronil	Y=2883.13X-7782.74	0.998±0.13	Y=1369.38X-9492.76	0.998±0.31	8.0	8.1
Flazasulfuron	Y=10385.27X-36665.18	0.996±0.44	Y=1574.25X-3459.24	0.998±0.10	24.8	14.9
Flonicamid	Y=2694.12X-6448.52	0.996±0.25	Y=1103.94X-3031.77	0.998±0.31	12.0	-3.6
Fluazinam	Y=1822.32X-7309.60	0.995±0.56	Y=869.13X-853.85	0.995±0.62	13.8	-5.6
Flubendiamide	Y=356.26X-3124.69	0.996±0.33	Y=507.08X-4601.05	0.996±0.52	9.5	34.3
Flufenacet	Y=37390.97X-324153.67	0.995±0.76	Y=13762.93X-74551.37	0.996±0.30	40.2	-2.2
Flufenoxuron	Y=5029.39X-674.06	1.000±0.03	Y=1261.96X-2000.83	0.995±0.76	1.1	-19.2
Flumetsulam	Y=9640.78X-21821.42	0.999±0.05	Y=2062.42X-5242.88	0.995±0.93	13.0	-42.0
Flumioxazin	Y=367.07X-1872.82	0.997±0.78	Y=142.98X-819.76	0.999±0.03	32.4	-13.7
Fluometuron	Y=43104.40X-294270.42	0.995±0.34	Y=16739.53X-70720.35	0.997±0.35	-0.8	-11.4
Fluopicolide	Y=28073.25X-105587.24	0.995±0.27	Y=8116.07X-27332.72	0.997±0.26	25.1	-2.9
Fluoxastrobin	Y=20488.59X-53609.45	0.997±0.19	Y=5613.63X-9975.52	0.999±0.06	10.3	-20.5
Fluquinconazole	Y=3201.37X-14957.28	0.997±1.12	Y=10.38X+112.58	0.998±1.31	66.2	-15.8
Flusilazole	Y=14576.08X-59440.63	0.995±0.21	Y=3860.72X+11377.60	0.997±0.31	20.6	-21.0
Flutriafol	Y=11035.43X-35423.44	0.999±0.04	Y=4110.51X+782.73	0.999±0.03	-4.5	-20.6
Foramsulfuron	Y=3182.55X+3656.31	0.999±0.07	Y=512.49X+1667.83	0.999±0.01	24.5	-17.1
Fosthiazate	Y=86438.09X-170139.08	0.996±0.26	Y=26212.14X-58837.72	0.997±0.31	16.6	-4.3
Fuberidazol	Y=63307.15X+12396.81	0.999±0.07	Y=24182.97X-6283.31	0.999±0.14	-7.8	-17.8
Furalaxyl	Y=100477.52X-384530.03	0.995±0.62	Y=32324.21X-50394.58	0.999±0.13	23.9	-16.1
Furathiocarb	Y=27253.50X-77607.46	0.996±0.20	Y=9002.19X-26266.16	0.996±0.57	22.6	-21.6
Halofenozide	Y=2511.68X-6772.92	0.998±0.10	Y=1055.46X-7046.04	0.998±0.27	-1.9	6.4
Hexaconazole	Y=8938.80X-14913.55	0.999±0.05	Y=2030.63X-142.06	0.998±0.14	18.2	-24.6
Hexaflumuron	Y=1133.96X-279.11	0.999±0.04	Y=345.76X+647.16	0.999±0.02	-12.4	-34.8
Hexythiazox	Y=13478.78X-1563.30	1.000±0.01	Y=4172.11X+8076.83	0.999±0.06	-2.7	-15.7
Hydramethylnon	Y=27372.81X-39423.13	0.998±0.16	Y=8623.75X-1313.99	0.998±0.22	9.0	-11.0
Imazalil	Y=9275.52X-13050.83	0.999±0.06	Y=2141.59X+237.18	0.999±0.11	14.6	-32.1
Imidacloprid	Y=6305.20X-11833.43	0.998±0.10	Y=1745.73X-3872.54	0.999±0.08	21.8	-30.2
Indoxacarb	Y=1898.50X-7249.72	0.995±0.21	Y=488.12X-146.45	0.999±0.02	38.4	-18.9
Ipconazole	Y=21690.06X-23995.17	0.999±0.03	Y=4629.58X-5252.31	0.997±0.41	14.3	65.3
Iprovalicarb	Y=50110.50X-129998.54	0.996±0.20	Y=13686.27X-21876.04	0.999±0.07	11.2	-6.3
Isocarbophos	Y=27264.71X-79772.06	0.996±0.25	Y=9452.84X-10772.60	0.996±0.49	13.6	-16.0
Isofenphos-methyl	Y=4586.54X-858.30	0.999±0.06	Y=921.79X-929.32	0.998±0.26	20.2	-32.5
Isoprothiolane	Y=92790.70X-371899.79	0.995±0.49	Y=25943.14X-41150.50	0.999±0.04	30.1	-1.7
Isoxaben	Y=79128.53X-105713.04	0.996±0.26	Y=21692.66X+7381.92	0.996±0.51	13.5	-15.5
Ivermectin B1a	Y=42.17X+87.97	0.997±0.15	Y=12.14X+33.37	0.998±0.30	35.1	-9.3
Kresoxim-methyl	Y=8600.74X-46875.34	0.996±0.44	Y=3068.05X-21566.72	0.999±0.03	17.3	-15.4

Lenacil	Y=36700.11X-82668.95	0.999±0.06	Y=1725.04X-6586.70	0.997±0.40	11.7	-65.7
Linuron	Y=9291.54X-57261.35	0.995±0.38	Y=3747.29X-1997.39	0.999±0.07	16.9	-10.9
Lufenuron	Y=1734.19X+449.28	1.000±0.03	Y=433.21X-237.48	0.998±0.16	4.2	-21.6
Malaoxon	Y=60491.53X-72084.06	0.999±0.08	Y=17597.31X-3987.95	0.997±0.37	9.9	-18.6
Malathion	Y=13932.53X-58420.86	0.996±0.39	Y=3737.87X-17489.09	0.999±0.10	23.8	-27.4
Mandipropamid	Y=20423.09X-28877.09	0.998±0.11	Y=5906.77X-4831.66	0.998±0.23	12.5	-13.2
Mecarbam	Y=23082.08X-142040.48	0.995±0.50	Y=8451.34X-13224.40	0.999±0.06	20.9	-14.1
Mepanipyrim	Y=18532.62X-65721.10	0.995±0.32	Y=5721.86X-8178.07	0.998±0.25	21.5	-18.2
Mesosulfuron-methyl	Y=5573.02X+4784.55	1.000±0.02	Y=999.85X-4274.27	0.998±0.22	-3.2	-5.6
Metaflumizone	Y=578.34X-611.57	0.997±0.18	Y=155.61X+436.58	0.998±0.25	22.5	-14.9
Metalaxyl	Y=67521.03X-151483.18	0.997±0.15	Y=17548.13X-51008.23	0.999±0.00	13.6	-22.9
Metamitron	Y=6686.34X-12826.12	0.996±0.23	Y=3561.51X-9406.06	0.999±0.14	-8.8	-15.7
Metazachlor	Y=60893.98X-78815.99	0.998±0.11	Y=18357.35X-10204.14	0.999±0.10	14.2	-13.6
Metconazole	Y=15782.85X-79624.22	0.995±0.19	Y=4240.93X-16935.97	0.999±0.07	30.2	-28.6
Methabenzthiazuron	Y=67118.30X+264681.49	0.997±0.40	Y=31554.60X-52963.60	0.998±0.16	-21.9	-19.5
Methacrifos	Y=2126.19X-10006.30	0.996±0.54	Y=410.16X+702.36	0.995±0.80	9.1	19.9
Methamidophos	Y=8400.38X+8256.58	1.000±0.01	Y=9428.46X-2186.47	0.997±0.46	44.9	-43.0
Methidathion	Y=11180.51X-48851.37	0.995±0.33	Y=3741.07X-19461.90	0.999±0.02	18.3	-16.6
Methiocarb	Y=15294.40X-70284.09	0.995±0.24	Y=6311.13X-40828.64	0.999±0.17	6.1	-20.1
Methomyl	Y=28811.78X-19584.28	0.997±0.21	Y=11828.44X-30032.57	0.998±.18	-13.1	-15.0
Methoprotryne	Y=55473.91X-41315.10	0.998±0.11	Y=14883.37X+8482.26	0.998±0.15	6.5	-17.8
Methoxyfenozide	Y=37911.15X-122426.02	0.996±0.17	Y=10903.17X-20586.73	0.999±0.13	10.1	-23.1
Metobromuron	Y=8989.11X-16589.26	0.998±0.11	Y=2796.52X-6737.89	0.999±0.08	0.1	-17.4
Metolachlor	Y=89747.12X-130452.23	0.997±0.17	Y=22694.19X-39763.11	0.999±0.09	13.9	-21.4
Metrafenon	Y=18618.95X-56469.69	0.996±0.23	Y=5116.74X+2195.15	0.998±0.24	12.4	-17.7
Metsulfuron-methyl	Y=6932.71X-8829.15	0.998±0.14	Y=1477.94X+399.41	0.999±0.09	21.4	-18.1
Mevinphos	Y=14012.30X-22616.00	0.998±0.12	Y=4783.43X-13398.54	0.999±0.06	8.3	-17.2
Mexacarbate	Y=77347.76X+29281.59	0.998±0.11	Y=31835.52X-37810.63	0.999±0.08	9.2	57.5
Molinate	Y=4830.04X-6509.43	0.999±0.06	Y=1764.72X+1016.35	0.995±0.48	6.9	-1.3
Monocrotophos	Y=6263.11X-3090.16	0.999±0.05	Y=4375.29X-8370.87	0.999±0.01	-4.8	-16.2
Moxidectin	Y=255.62X-189.07	0.999±0.07	Y=57.08X-26.96	0.999±0.02	7.2	-11.3
Myclobutanil	Y=14872.33X-6619.18	1.000±0.02	Y=3653.64X+2713.57	0.998±0.11	12.1	-5.0
Nicosulfuron	Y=7795.83X-8793.08	0.999±0.09	Y=965.10X+18.03	0.999±0.02	18.7	31.8
Nitenpyram	Y=4945.67X-5039.12	0.995±3.09	Y=3402.86X-4721.35	0.999±0.07	69.2	-27.8
Novaluron	Y=3140.02X-2108.16	0.999±0.02	Y=1013.98X+2657.77	0.999±0.05	2.9	-23.1
Omethoate	Y=28587.96X+73948.46	1.000±0.00	Y=14718.15X-11425.13	0.999±0.12	38.9	-14.9
Oxadiazon	Y=1571.96X-890.07	0.999±0.08	Y=498.20X+1211.95	0.999±0.05	5.2	-26.1
Oxamyl	Y=38498.35X+42639.83	1.000±0.03	Y=21460.22X-9996.30	0.996±0.04	17.9	-18.7
Oxasulfuron	Y=28675.22X-24638.22	0.998±0.11	Y=8174.40X+17920.52	0.999±0.07	3.1	-16.3
Paclobutrazol	Y=21158.85X-75207.66	0.997±0.14	Y=6041.02X-27423.82	0.999±0.03	16.7	-17.2
Penconazole	Y=15852.40X-60724.86	0.996±0.16	Y=5186.86X-4134.20	0.998±0.18	13.9	-18.2
Pencycuron	Y=70613.04X-130825.61	0.996±0.25	Y=19904.65X+308.53	0.999±0.08	23.2	-10.7
Pendimethalin	Y=8058.62X-13561.61	0.998±0.09	Y=2227.02X-712.77	0.998±0.29	10.5	-15.8
Phenmedipham	Y=43935.76X-173879.63	0.995±0.33	Y=15140.28X+56174.00	0.997±0.44	11.7	51.2
Phenthoate	Y=23053.56X-152637.84	0.996±0.49	Y=7431.17X-42026.82	0.997±0.24	25.3	-14.8
Phosalone	Y=5727.62X-13811.62	0.998±0.12	Y=1800.80X-4967.78	0.998±0.19	13.4	-25.2
Phosmet	Y=36473.92X-68063.34	0.999±0.05	Y=11933.60X+10619.62	0.996±0.46	9.0	99.2
Phosphamidone	Y=23538.83X+16048.83	1.000±0.02	Y=6667.79X+11191.04	0.999±0.06	-2.4	42.1
Phoxim	Y=26787.22X-73571.80	0.997±0.12	Y=9079.92X-27436.11	0.999±0.03	10.3	-14.2
Picolinafen	Y=30291.64X-91303.05	0.997±0.19	Y=7923.83X-16329.08	0.997±0.27	12.1	-20.2
Picoxystrobin	Y=90042.75X-287722.20	0.995±0.54	Y=24510.78X-1454.61	0.998±0.25	20.7	-26.9
Pirimicarb	Y=110209.72X+20472.44	0.997±0.22	Y=34265.18X+39742.84	0.995±0.72	7.9	-19.6
Pirimiphos-methyl	Y=33657.80X-47551.13	0.999±0.07	Y=9504.00X-5799.35	0.997±0.30	12.8	-17.6
Prochloraz	Y=23315.51X-55445.31	0.997±0.20	Y=5831.78X-15852.78	0.999±0.04	21.2	-3.2
Profenofos	Y=14140.73X-30174.08	0.999±0.05	Y=3964.38X-689.00	0.999±0.05	5.1	-20.2
Promecarb	Y=30174.08X-80640.33	0.997±0.16	Y=9687.43X-23292.10	0.999±0.08	10.4	-18.1
Prometon	Y=77152.47X-107272.59	0.997±0.18	Y=23047.33X-4773.74	0.999±0.10	8.2	-18.0
Propamocarb	Y=102485.56X+15992.25	0.997±0.20	Y=36849.53X+138008.16	0.999±0.14	-15.4	-11.0
Propaquizafop	Y=19255.53X-17045.91	0.998±0.15	Y=5199.86X-1130.54	0.999±0.04	18.6	-22.7
Propargite	Y=44984.24X-73216.70	0.997±0.17	Y=11038.91X-9023.22	0.998±0.20	14.9	-18.5
Propetamophos	Y=4755.04X-12286.11	0.995±0.41	Y=1120.68X-3293.24	0.996±0.65	11.7	-16.4
Propham	Y=5467.25X-435.10	1.000±0.02	Y=2375.39X+2184.55	0.998±0.15	4.4	-6.3

Propiconazole	Y=2791.80X-22511.14	0.995±0.16	Y=683.80X-5006.37	0.999±0.02	16.7	-30.9
Propoxur	Y=63129.17X-47098.67	0.999±0.07	Y=21155.27X+10123.14	0.995±0.56	8.8	-9.8
Propyzamid	Y=12377.87X-81835.70	0.996±0.57	Y=4616.50X-15571.06	0.999±0.04	26.3	34.4
Proquinazid	Y=19455.43X-20681.79	0.999±0.06	Y=5086.10X+437.88	0.997±0.37	19.4	-10.2
Prosulfocarb	Y=77757.12X-136847.18	0.996±0.28	Y=25003.83X+3774.41	0.996±0.62	13.3	-15.7
Pymetrozine	Y=6367.97X-53092.03	0.997±0.66	Y=9326.09X-32842.71	0.997±0.40	14.9	-19.1
Pyracarbolid	Y=91409.85X-180317.55	0.995±0.34	Y=33181.55X-28188.78	0.979±3.58	56.9	-10.5
Pyraclostrobin	Y=27441.25X-69312.64	0.998±0.12	Y=7380.43X-24523.24	0.995±0.77	11.1	-24.9
Pyridaben	Y=69532.60X-180779.79	0.995±0.39	Y=18991.85X+4092.71	0.998±0.10	17.6	-21.2
Pyridat	Y=270.58X+277.33	1.000±0.02	Y=710.00X+2955.56	0.995±1.65	-22.4	-42.8
Pyrimethanil	Y=8431.53X-14942.41	0.997±0.16	Y=2987.54X+2798.22	0.995±1.37	20.3	-12.2
Pyriproxyfen	Y=75157.85X-170279.04	0.995±0.30	Y=22390.14X-18528.96	0.999±0.09	15.3	-24.4
Quinalphos	Y=7483.65X-46965.62	0.996±0.35	Y=2495.59X-1278.94	0.997±0.39	22.9	-13.0
Quinmerac	Y=36262.04X-69816.71	0.996±0.24	Y=19730.24X-27620.06	0.997±0.36	9.5	-11.5
Quinoclamin	Y=5467.97X-23973.90	0.995±0.46	Y=2572.45X-6621.41	0.999±0.06	2.7	51.7
Quinoxyfen	Y=14959.72X-21638.99	0.999±0.07	Y=4399.74X-1288.51	0.998±0.25	6.8	-21.5
Rimsulfuron	Y=5811.17X+3829.73	1.000±0.02	Y=554.17X+1973.10	0.999±0.01	27.7	61.9
Rotenone	Y=3397.93X-20374.90	0.995±0.47	Y=933.42X+2399.79	0.998±0.13	15.8	-20.1
Secbumeton	Y=68232.31X-115003.37	0.997±0.19	Y=20983.73X-13650.86	0.998±0.24	10.1	-16.7
Silthiopham	Y=20700.10X-60244.67	0.995±0.62	Y=6183.27X-13579.41	0.999±0.14	34.3	-22.4
Spinosyn A	Y=19593.75X-16933.70	0.999±0.07	Y=6007.26X-1565.56	0.999±0.01	8.1	-22.1
Spinosyn D	Y=3057.66X+576.22	1.000±0.03	Y=856.47X+194.10	0.999±0.02	4.5	-25.9
Spirodiclofen	Y=13381.29X-15673.78	0.999±0.08	Y=3249.61X-929.35	0.999±0.01	14.5	-18.6
Spiromesifen	Y=14041.43X-62845.41	0.996±0.49	Y=4271.24X-10752.26	0.995±0.70	16.4	-18.4
Spirotetramat	Y=7870.42X-29081.79	0.997±0.13	Y=1979.45X-269.81	0.998±0.20	16.8	-11.7
Sulfentrazone	Y=676.42X-102.74	0.999±0.07	Y=4201.57X-7667.58	0.999±0.11	18.2	35.4
Tebuconazole	Y=16113.40X-59650.81	0.999±0.02	Y=14562.24X+5158.94	0.999±0.06	-8.2	63.3
Tebufenozid	Y=48116.12X-158350.58	0.996±0.45	Y=3283.99X+3201.06	0.999±0.01	23.1	-81.5
Tebufenpyrad	Y=11216.48X-35616.42	0.996±0.18	Y=20983.71X-56208.19	0.998±0.18	13.3	50.5
Tebuthiuron	Y=62398.97X-89604.14	0.998±0.10	Y=556.76X-19410.27	0.998±0.29	-0.1	-97.8
Teflubenzuron	Y=525.90X-9266.08	0.997±0.55	Y=2220.87X+7189.32	0.999±0.04	27.3	47.6
Tepraloxydim	Y=4564.85X-17936.84	0.995±0.47	Y=691.08X+2227.93	0.995±1.41	12.5	70.6
Terbufos	Y=2213.66X-4066.91	0.997±0.14	Y=1072.47X-5525.00	0.996±0.22	18.6	47.7
Tetraconazole	Y=4126.61X-39705.13	0.996±0.49	Y=20688.03X+6208.72	0.997±0.22	34.1	45.9
Thiabendazole	Y=56011.93X+170829.28	0.999±0.00	Y=16127.76X-15574.55	0.995±0.67	81.7	-40.4
Thiacloprid	Y=51294.22X-80637.06	0.997±0.16	Y=3740.91X-11474.51	0.999±0.02	19.7	-81.6
Thifensulfuron-methyl	Y=5516.16X+11482.28	1.000±0.01	Y=989.50X+4219.37	0.997±0.40	1.3	-11.0
Thiodicarb	Y=25493.66X-88726.75	0.998±0.04	Y=5621.77X-2380.64	0.998±0.07	10.5	-13.4
Thiofanox	Y=605.91X-5040.59	0.995±0.13	Y=523.95X-973.57	0.997±3.98	3.7	-25.9
Tolclofos-methyl	Y=2599.62X-3728.14	0.998±0.15	Y=863.37X-1118.33	0.998±0.24	16.6	-14.0
Tolylfluanide	Y=1503.77X-9243.32	0.995±0.27	Y=505.93X-3039.82	0.999±0.11	-16.0	-6.7
Tralkoxydim	Y=7857.54X-2904.00	0.999±0.07	Y=1924.84X+3270.99	0.998±0.11	15.7	-11.8
Triadimefon	Y=9600.66X-16824.32	0.999±0.07	Y=2508.33X-4963.86	0.997±0.44	10.1	29.1
Triadimenol	Y=4084.88X-23398.49	0.996±0.28	Y=846.90X-3300.04	0.999±0.04	24.1	90.2
Triasulfuron	Y=3173.18X+172.11	1.000±0.03	Y=678.19X+2296.16	0.998±0.15	5.4	-24.7
Triazophos	Y=79542.57X-341802.16	0.996±0.40	Y=23700.39X-72482.25	0.999±0.01	20.7	-17.7
Trichlorfon	Y=384.91X-2748.20	0.997±0.84	Y=306.85X-1533.12	0.996±0.51	-35.5	-12.4
Tricyclazol	Y=55249.34X-49962.94	0.999±0.08	Y=17826.54X-9896.36	0.997±0.11	11.4	-30.1
Trietazin	Y=16260.98X-92047.99	0.996±0.58	Y=5538.91X-12738.30	0.999±0.03	33.7	-12.9
Trifloxystrobin	Y=43779.89X-182119.97	0.995±0.42	Y=13313.64X-19219.19	0.996±0.63	14.6	-18.4
Triflumizol	Y=60018.09X-125484.90	0.998±0.10	Y=17153.47X-4721.27	0.998±0.18	74.2	53.1
Triflumuron	Y=7802.31X-47120.97	0.995±0.33	Y=2459.94X-12936.27	0.999±0.01	19.1	-16.2
Trimethacarb	Y=51080.00X-155919.59	0.995±0.25	Y=20621.97X-43430.88	0.996±0.55	19.8	-14.7
Triticonazole	Y=7612.81X-28689.15	0.995±0.22	Y=1953.53X-2726.47	0.998±0.17	23.0	-25.4
Uniconazole-P	Y=13557.79X-32518.57	0.998±0.12	Y=3409.88X-24609.77	0.999±0.04	21.1	40.2
Vamidothion	Y=71821.44X+19084.24	0.999±0.09	Y=20118.35X+7994.29	0.999±0.11	12.9	-32.5
Zoxamide	Y=28745.46X-103718.42	0.995±0.25	Y=7885.35X+6118.39	0.997±0.45	20.0	-14.1

Table S3: Wavelengths, linear regression equation, coefficient of regression, and LOQ of the studied metals and metalloids.

Element	Wavelength (nm)	Linear regression equation	R^2 ± RSD%	LOQ mg kg^{-1}
Ag	328.068	Y=16.2X+53.8	1,000 ± 0.01	0.005
Al	396.152	Y=43.0X+339.3	0,996 ± 0.17	0.010
As	193.696	Y=3.1X+33.5	0,997 ± 0.13	0.005
B	249.678	Y=6.7X+36.2	0,997 ± 0.13	0.005
Ba	455.403	Y=2946X+26178.1	0,997 ± 0.15	0.005
Be	234.861	Y=59.7X+506.8	0,997 ± 0.14	0.005
Ca	396.847	Y=4552X-130789.3	1,000 ± 0.01	0.050
Cd	214.439	Y=12.7X+136.1	0,997 ± 0.14	0.005
Co	230.786	Y=5.7X+64.6	0,997 ± 0.15	0.005
Cr	267.716	Y=20.3X+144.5	0,997 ± 0.15	0.005
Cu	327.395	Y=21.6X+171.0	0,997 ± 0.14	0.005
Fe	238.204	Y=15.9X-0.7	0,997 ± 0.12	0.050
Hg	253.652	Y=2.0X+52.4	0,996 ± 0.15	0.050
Li	610.365	Y=110.3X-824.9	0,996 ± 0.12	0.050
Mg	279.553	Y=340X+3220.5	0,996 ± 0.18	0.005
Mn	257.610	Y=107X+1039.9	0,997 ± 0.16	0.005
Mo	281.615	Y=9.6X+86.7	0,997 ± 0.14	0.005
Ni	231.604	Y=1.6X+28.0	0,998 ± 0.07	0.010
Pb	283.305	Y=0.9X+38.5	0,997 ± 0.03	0.050
Sb	187.052	Y=0.1X+9.7	0,998 ± 0.01	0.050
Se	196.026	Y=0.4X+12.3	0,997 ± 0.09	0.010
Sn	189.925	Y=0.2X+4.0	0,996 ± 0.01	0.005
Sr	407.771	Y=76.37X+85891	0,997 ± 0.15	0.005
Te	214.820	Y=0.3X+18.2	0,996 ± 0.17	0.050
Ti	334.941	Y=127X+789	0,997 ± 0.14	0.005
V	309.310	Y=11.5X+17.0	0,997 ± 0.14	0.010
Zn	213.857	Y=10.0X+55.6	0,997 ± 0.16	0.005
